# M1 macrophage-derived exosomal miR-20b promotes radiosensitization via CCND1 in HPV^+^ HNSCC

**DOI:** 10.3389/fonc.2025.1693487

**Published:** 2025-11-27

**Authors:** Huan Liu, Siwei Zhang, Zengchen Liu, Tingdan Gong, Siyu Duan, Tianyang Liu, Fangjia Tong, Wanlin Li, Shuang Pan, Lanlan Wei

**Affiliations:** 1 The First Affiliated Hospital of Harbin Medical University, Harbin Medical University, School of Stomatology, Harbin, Heilongjiang, China; 2 National Clinical Research Center for Infectious Diseases, The Third People’s Hospital of Shenzhen, The Second Hospital Affiliated to Southern University of Science and Technology, Shenzhen, Guangdong, China; 3 Department of Microbiology, Harbin Medical University, Harbin, Heilongjiang, China; 4 Department of Pharmacology, Northwestern University Feinberg School of Medicine, Chicago, IL, United States

**Keywords:** HPV, HNSCC, exosome, miR-20b, radiotherapy

## Abstract

**Background:**

Human papillomavirus (HPV) is a significant risk factor for head and neck squamous cell carcinoma (HNSCC). HPV positive (HPV^+^) HNSCC is more sensitive to radiotherapy and has a better prognosis than HPV negative (HPV^-^) HNSCC. M1 macrophages not only enhance the radiosensitivity of HPV+ HNSCC, but the M1 macrophages derived exosmoes (M1 exos) also possess anti-tumor activity. However, the role of M1 exos in the radiosensitivity of HNSCC remains unclear.

**Materials and methods:**

HPV status and macrophage infiltration levels in 25 HNSCC tissues were evaluated by immunohistochemistry (IHC). M1 macrophages were induced and cultured *in vitro*, and exosomes were extracted through differential ultracentrifugation. The effect of M1 exos on the radiosensitivity of HPV^+^ HNSCC was assessed via an *in vitro* co-culture system. The expression level of γ-H2AX was assessed by immunofluorescence. The levels of miR-20b in HNSCC were analyzed using multicenter data (from TCGA and GEO databases), along with their correlation to radiosensitivity and prognosis. Cellular experiments demonstrated that overexpressing miR-20b significantly enhanced radiosensitivity in HPV+ HNSCC. Bioinformatic and experimental validation identified CCND1 as a target of miR-20b.

**Results:**

In HPV^+^ HNSCC, M1 macrophages were highly infiltrated and played a crucial role in improving the therapeutic effect of HPV^+^ HNSCC. M1 exos infiltrated HPV^+^ HNSCC, increasing their sensitivity to radiation. Meanwhile, M1 macrophages exhibited higher miR-20b levels than M2 macrophages, and the radiosensitivity of HPV^+^ HNSCC was significantly increased by transfecting them with a miR-20b mimic. Functional analysis of target genes, CCND1 as a key gene through which miR-20b enhanced radiosensitivity in HPV^+^ HNSCC.

**Conclusion:**

In this study, our results suggest that M1 exos, enriched with miR-20b, regulate the DNA damage repair pathway in tumor cells by targeting CCND1, enhancing the radiosensitivity of HPV^+^ HNSCC.

## Introduction

Head and neck cancer, the sixth most prevalent cancer worldwide, presents in various anatomical locations within the head and neck region. More than 890,000 new cases were confirmed, and approximately 450,000 deaths were reports in 2022 (1, 2). Approximately 90% of head and neck malignancies are classified as squamous cell carcinomas, abbreviated as HNSCC. Human papillomavirus (HPV) is one of the important causes of HNSCC. According to the status of HPV infection, HNSCC is divided into HPV positive (HPV^+^) HNSCC and HPV negative (HPV^-^) HNSCC. Compared to HPV^-^ HNSCC, HPV^+^ HNSCC is a special heterogeneous tumor with unique molecular and clinical features. HPV^+^ HNSCC typically presents with smaller tumors, demonstrates a stronger response to radiotherapy, and exhibits a tumor microenvironment characterized by enhanced immune crosstalk and upregulation of associated pathways (3–5). However, as one of the primary methods of HNSCC, the mechanism by which HPV regulates the radiosensitivity of tumor cells still requires further exploration.

Tumor-associated macrophages (TAMs) represent the predominant cell type within the tumor microenvironment, constituting roughly 50% of its cellular composition (6–8). They are essential to the processes of tumor initiation and progression. TAMs originate from circulating monocytes. Following stimulation, they polarize to M1 macrophages, which are characterized by anti-tumor functions, and M2 macrophages, which are associated with pro-tumor activities (9). The extensive infiltration of M1 macrophages induces tumor cell death by secreting cytotoxic molecules, such as inflammatory mediators, and by recruiting cytotoxic immune cells, including T cells, thereby enhancing the overall antitumor immune response.

During tumor radiotherapy, M1 macrophages enhance treatment efficacy by amplifying the intrinsic DNA damage response in tumor cells. HPV^+^ HNSCC recruits M1 macrophages through the release of interleukin-6 and miR-9, improving radiosensitivity (10, 11). Studies have found M1 macrophage-derived exosomes (M1 exos) accumulate in the tumor microenvironment, reducing the infiltration of immunosuppressive cells, enhancing the therapeutic sensitivity (12, 13). Engineered M1 exos modify the tumor microenvironment, promote the repolarization of M2 macrophages, enhance phagocytosis, and act as sensitizers for radiotherapy (14). Exosomes are lipid bilayer vesicles characterized by a diameter between 30 and 200 nanometers (15), consist of various bioactive molecules, including microRNAs (miRNAs), proteins, and mRNAs (16, 17). MiRNAs are small, single-stranded RNA molecules ranging in length from 19 to 25 nucleotides, which act as significant promoters or suppressor of tumor progression (18). Researchers have found that miR-20b-5p inhibits the progression of thyroid and bladder cancers by modulating the MAPK-ERK signaling pathway and targeting key cell cycle-related proteins (19, 20). Researches have shown that miRNA-20b is the miRNA most significantly correlated with HPV16. In oropharyngeal cancer, miR-20b is higher and associated with a favorable prognosis in HNSCC (21, 22). Additionally, miR-20b can enhance tumor radiosensitivity or chemosensitivity, then improving prognosis when combined with immune checkpoint inhibitors (22). However, whether miR-20b contributes to the radiosensitizing effect of M1 macrophages in HPV^+^ HNSCC is still unclear.

In this study, we found that M1 exos, enriched with miR-20b, enhanced radiosensitivity by targeting and inhibiting the key factor CCND1 in the DNA damage repair pathway in HPV^+^ HNSCC. This research provides a new theoretical basis and experimental evidence for elucidating the radiosensitization mechanism of HPV^+^ HNSCC.

## Material and methods

### Patients samples

The samples were collected from 25 patients diagnosed with HNSCC at Shenzhen Third People’s Hospital from 2021 to 2023. The research ethics committee of Shenzhen Third People’s Hospital approved this study in accordance with the Declaration of Helsinki (2021-056). Clinical information and written informed consent were obtained from all participants involved in the study. The clinical characteristics of the patients were shown in **Supplementary Table S1**.

### Cell lines

The HNSCC cell lines used in this study included SCC47 (HPV^+^), kindly provided by Dr. Henning Willers (Harvard University, USA); SCC90 (HPV^+^), obtained from the American Type Culture Collection; and CAL27 (HPV^-^), a generous gift from Professor Songbin Fu (Harbin Medical University, China). All cells were cultured in high-glucose DMEM (ThermoFisher, C11995500BT) supplemented with 10% fetal bovine serum (FBS) (GIBCO, 10099141C) and 1% penicillin-streptomycin solution (PS) (P1400). The cells were tested for mycoplasma contamination, and no mycoplasma was detected. The cell lines were cultured in a humidified incubator at 37 °C with 5% CO_2_.

### Macrophage differentiation and polarization

The human monocyte cell line THP-1 was obtained from the American Type Culture Collection and cultured in RPMI 1640 medium (ThermoFisher, C11875500BT) supplemented with 10% FBS and 1% PS. All cells were maintained at 37 °C in a 5% CO_2_ atmosphere. Macrophage polarization was performed as previously described (10). Briefly, THP-1 cells were seeded in 12-well plates at a density of 5 × 10^5^ cells per well and treated with 100 ng/mL phorbol 12-myristate 13-acetate (PMA) (MCE, HY18739) to induce macrophage differentiation. After 24 h, 100 ng/mL lipopolysaccharide (LPS) (Beyotime, S1732) and 20 ng/mL interferon-gamma (IFN-γ) (Beyotime, P5664) were added for an additional 48 h to induce M1 macrophages. To obtain M1 exos without the influence of FBS-derived exosomes, the FBS was ultracentrifuged at 120,000 g for 20 h prior to use.

### Exosome isolation and characterization

The M1 exos were isolated from the cell supernatant using differential ultracentrifugation (11). Briefly, the culture supernatant of M1 macrophages was collected and centrifuged at 200 g for 15 min, followed by centrifugation at 2,000 g for 20 min. This was followed by ultracentrifugation at 10,000 g for 30 min, and finally, centrifugation at 100,000 g for 70 min. The exosomes were then re-suspended in 50-100 μL of pre-cooled PBS and stored at -80 °C. All ultracentrifugation procedures were conducted using a Beckman Coulter centrifuge. The temperature during all centrifugation steps was maintained at 4 °C, and the operations were performed on ice.

Particle size and concentration were analyzed using nanoparticle tracking analysis (NTA) with a Zetaview instrument from Particle Metrix (Germany). The isolated exosomes (10 μL) were placed on a copper mesh for 5 to 10 min, and the excess liquid was absorbed with filter paper in preparation for transmission electron microscopy (TEM) analysis. The samples were then visualized using a Hitachi HT7700 transmission electron microscope.

### Western blotting

The cells and M1 exos were collected for Western blotting (23). The whole cell lysates and M1 exos lysates were extracted using radioimmunoprecipitation assay (RIPA) (Solarbio, R0010-100) containing a proteinase inhibitor cocktail. The proteins were isolated using SDS-PAGE gel and subsequently transferred to a polyvinylidene fluoride membrane (PVDF) (Roche, 3010040001). The membranes were incubated at room temperature with 5% skim milk for 1 h. The CD81 antibody (Proteintech, 66866-1-Ig), TSG101 antibody (Abcam, 67381-1-Ig) and calnexin antibody (Proteintech, 10427-2-AP) were incubated at 4 °C overnight. Then, secondary antibodies were incubated according to the species of the primary antibody. Finally, the membranes were incubated with ECL chemiluminescent solution for 1–2 min, the liquid was absorbed with filter paper, and the WB membranes were encapsulated with plastic film and exposed and photographed using a chemiluminescence imaging system. The experiment was repeated three times.

### M1 exos tracking

In order to verify whether the exosomes were taken up by SCC090 cells, the M1 exos were labeled with a green fluorescent dye (PKH67, Sigma-Aldrich) according to the manufacturer’s instructions. These labeled exosomes were then incubated with SCC090 cells at 37 °C for 2 h in the dark. The results were observed using a Zeiss LSM 980 confocal microscope.

### Immunohistochemistry

Immunohistochemical staining of the tissue was performed using the method described previously (10). Briefly, tissues were fixed in 4% paraformaldehyde, paraffin-embedded, and sectioned to a thickness of 4 μm. Endogenous peroxidase activity was blocked with 3% hydrogen peroxide for 30 min, and goat serum (Bioss, C01-03001) was applied for 15 min. Sections were stained overnight at 4 °C with antibodies against iNOS (1:100; Proteintech, 80517-1-RR), CD163 (1:200; Proteintech, 16646-1-AP), and p16 (1:150; ZSGB-Bio, ZM-0205), followed by incubation with a two-step IHC reagent (ZSGB-Bio, PV9000). The results were evaluated by two experienced pathologists in a double-blinded manner and graded according to previous research. Scores were assigned based on the proportion of positively stained cells and the intensity of staining: 0 (no positive cells), 1 (< 10% positive cells), 2 (10-50% positive cells), and 3 (> 50% positive cells). The intensity of staining was evaluated using a defined scale: 0 indicates no staining, 1 represents weak staining (light yellow), 2 denotes medium staining (tan), and 3 signifies strong staining (brown). The staining index was calculated by multiplying the staining intensity score by the proportional score. Staining indices of 0, 1, 2, 3, 4, 6, and 9 were utilized for the assessment of the IHC.

### RNA scope

Paraffin tissue sections were processed using the RNAscope^®^ 2.5 HD Detection Kit-BROWN (ACD, 322310) in accordance with the manufacturer’s instructions.

### Cell radiation assay

The HNSCC cell lines, cultured in a 12-well plate, were co-cultured with various treatments for 24 h. A single dose of 2 Gy of 225 kV X-rays was delivered to cells using an RadSource RS2000 (US) irradiator at a dose rate of 0.0308 Gy. After 24 h, the cells were fixed with 10% neutral formaldehyde for immunofluorescence staining of γ-H2AX.

### Immunofluorescence staining

The HNSCC cell lines were fixed in 10% neutral formaldehyde for 15 min, permeabilized with 0.5% Triton X-100, and blocked with 5% goat serum for 30 min. Rabbit anti-human γ-H2AX (1:200; Abcam, ab81299) was incubated overnight at 4 °C. After washing with PBS three times, the cells were incubated with Alexa Fluor 488 goat anti-rabbit IgG (H+L) (1:200; Proteintech Group, RGAR002) for 1 h at 37 °C in the dark, followed by three washes with PBS. Finally, the samples were stained with DAPI (Beyotime, P0131) to visualize the nuclei. Sections were imaged using a Zeiss LSM 980 confocal microscope.

### Cell transfection

The HNSCC cell lines in the logarithmic growth phase were seeded in 6-well plates at 5 × 10^5^ cells per well. The hsa-miR-20b-5p mimic, along with corresponding negative controls (50 nM, Abm, MIH01532), were transfected into the HNSCC cell lines using Lipofectamine 2000 (Invitrogen, 11668030) according to the manufacturer’s instructions. Opti-MEM™ I medium (GIBCO, 31985070) was used as the dilution reagent. After 6 h, the transfection reagent was replaced with complete medium, and the cells were cultured for an additional 24 h for subsequent functional experiments.

### Cell-viability assay

HNSCC cell lines were seeded in 96-well plates at a density of 2×10^5^ cells/mL. After sufficient attachment, the cells were transfected with miR-20b mimics, miR-NC (negative control), or left untransfected as a control, following the “Cell transfection” protocol. Subsequently, the cells were exposed to 0 Gy or 2 Gy of irradiation. After 24 h, cell viability was assessed by incubating the cells with CCK-8 solution (GOONIE, cck8-100-120) for 30 min, and absorbance was measured at 450 nm using a microplate reader.

### Quantitative reverse transcription polymerase chain reaction

Total RNA was extracted from cells using Trizol^®^ (Invitrogen, 15596018CN). The RNA was reverse transcribed using the PrimeScript RT Reagent Kit (Takara, RR047A) along with specific miR-20b stem-loop primers. TB Green Premix Ex Taq (Takara, RR820A) was utilized for qRT-PCR. U6 was served as an endogenous control. GADPH was used as an internal control in CCND1 detection. All experiments were conducted in triplicate, and the data were analyzed using the 2^-ΔΔCt^ method. The miR-20b primer sequences were designed using miRNA Design V1.01. The forward and reverse primers are detailed in the **Supplementary Material****Supplementary Table S2**.

### Bioinformatics analysis

HNSCC data were downloaded from The Cancer Genome Atlas (TCGA) (https://portal.gdc.cancer.gov/), which included 421 HPV^-^ HNSCC samples and 97 HPV^+^ HNSCC samples. Macrophage infiltration and survival analyses were conducted using TIMER 2.0 (http://timer.cistrome.org/) (24). The completely response was defined as the disappearance of the tumor six months after radiotherapy; otherwise, it was classified as nonresponse. The macrophage miRNA-seq expression profile data were obtained from the Gene Expression Omnibus (GEO) (https://www.ncbi.nlm.nih.gov/geo/). MiRDB (https://mirdb.org/) was utilized to analyze the target genes of miR-20b, with a target score greater than 50 set as the cutoff value. The Database for Annotation, Visualization, and Integrated Discovery (DAVID) (https://david.ncifcrf.gov) was employed for Gene Ontology (GO) and Kyoto Encyclopedia of Genes and Genomes (KEGG) enrichment analyses. STRING (https://string-db.org) was used to analyze gene interaction relationships, which were visualized using Cytoscape software (v3.10.1). Gene enrichment results were plotted using an online platform for data analysis and visualization (https://www.bioinformatics.com.cn).

### Statistical analysis

GraphPad Prism 10 was utilized for data analysis. The results are presented as mean ± SD. The Student’s t-test or one-way ANOVA was employed to compare two or more independent groups. The Kaplan-Meier method was used for survival analysis. Pearson chi-squared tests were conducted to evaluate the correlation between two variables. Data were collected from a minimum of three independent experiments. Statistical significance was defined as *p* < 0.05.

## Results

### The infiltration of M1 macrophages increased in HPV^+^ HNSCC

**Figure 1:**
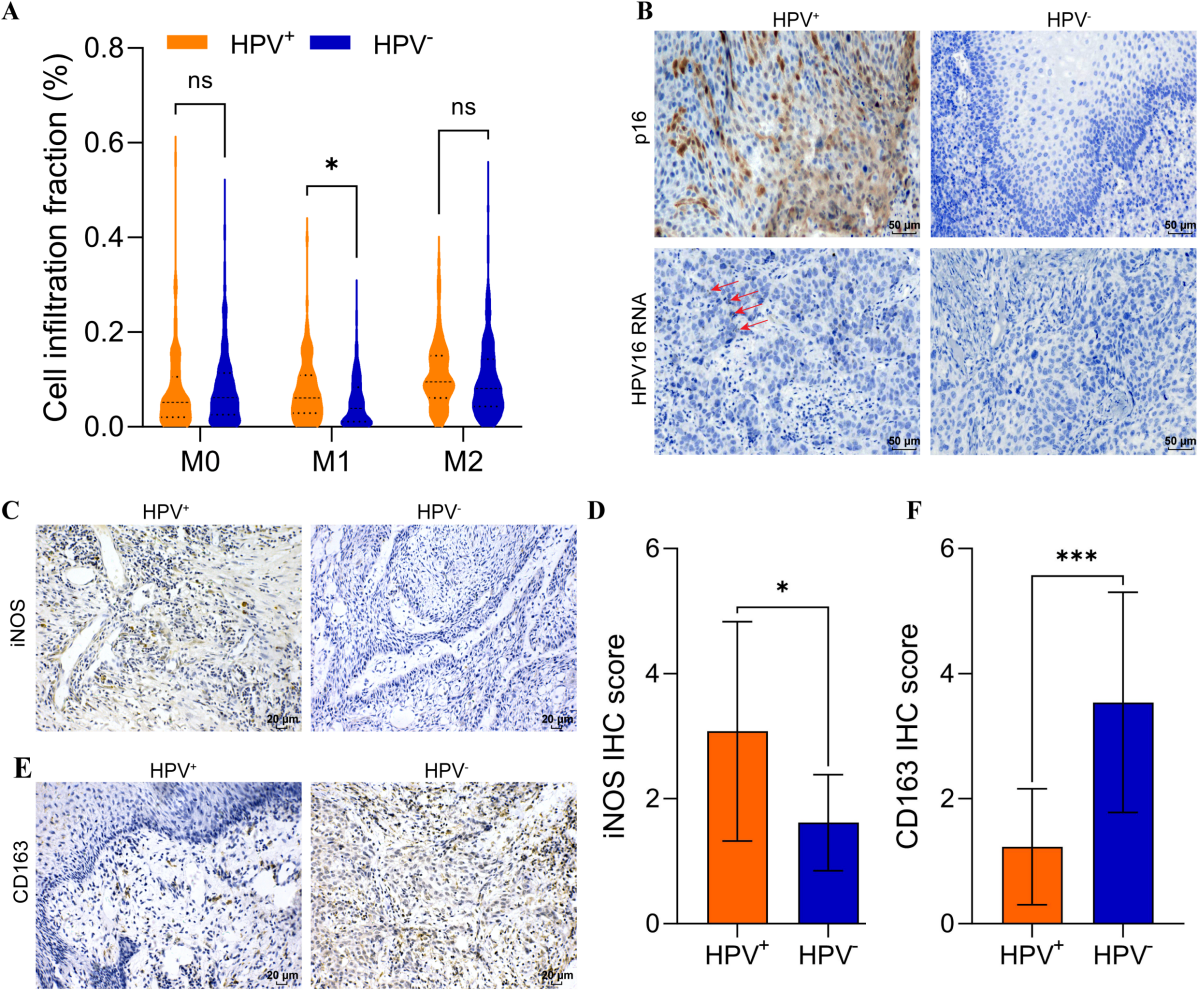
Elevated M1 macrophage infiltration in HPV^+^ HNSCC. **(A)** CIBERSORT-ABS analysis of M0, M1, M2 in HPV^+^ and HPV^-^ HNSCC tissues. **(B)** p16 was analyzed using IHC and RNA *scope* in HNSCC. Scal bar = 50 μm. **(C)** IHC analysis iNOS expression in HNSCC. Scal bar = 20 μm. **(D)** Quantification of iNOS for IHC. **(E)** IHC analysis CD163 expression in HNSCC. Scal bar = 20 μm. **(F)** Quantification of CD163 for IHC. ^*^P < 0.05, ^***^P < 0.001, ns, no significance.

We analyzed a cohort of 518 patients with HNSCC from the TCGA, which included 421 HPV^-^ HNSCC and 97 HPV^+^ HNSCC. Using macrophage infiltration abundances estimated by CIBSORT-ABS, we analyzed the association between HPV status and macrophage subtypes infiltration in HNSCC. No significant differences were observed in M0 or M2 macrophage levels between HPV^-^ HNSCC and HPV^+^ HNSCC. Interestingly, M1 macrophages were significantly elevated in HPV^+^ HNSCC compared to HPV^-^ HNSCC (*p* < 0.05) (**Figure 1A**).

Twenty-five samples were collected from patients with HNSCC, and the clinical characteristics were shown in **Supplementary Table S1**. The HPV status was assessed using IHC and RNA *scope* (**Figure 1B**). Then, the expression of iNOS (an M1 macrophage marker) and CD163 (an M2 macrophage marker) was analyzed using IHC to revealed distinct macrophage infiltration (**Figures 1C, E**). The results indicated that iNOS levels were significantly elevated in HPV^+^ HNSCC (*p* < 0.05) (**Figure 1D**). In contrast, CD163 levels were significantly increased in HPV^-^ HNSCC (*p* < 0.001) (**Figure 1F**). These results suggested that M1 macrophages were elevated in HPV^+^ HNSCC. Our histological findings were consistent with those from TCGA cohort. Analysis with the TIMER 2.0 database indicated that increased M1 macrophages infiltration in HPV^+^ HNSCC correlated with improved prognosis (**Supplementary Figures S1D-F**). Conversely, the infiltration levels of M0 and M2 macrophages did not impact the prognosis in either HPV^+^ HNSCC or HPV^-^ HNSCC (**Supplementary Figures S1A-C, G-I**).

### M1 macrophages enhanced the radiosensitivity of HPV^+^ HNSCC

**Figure 2:**
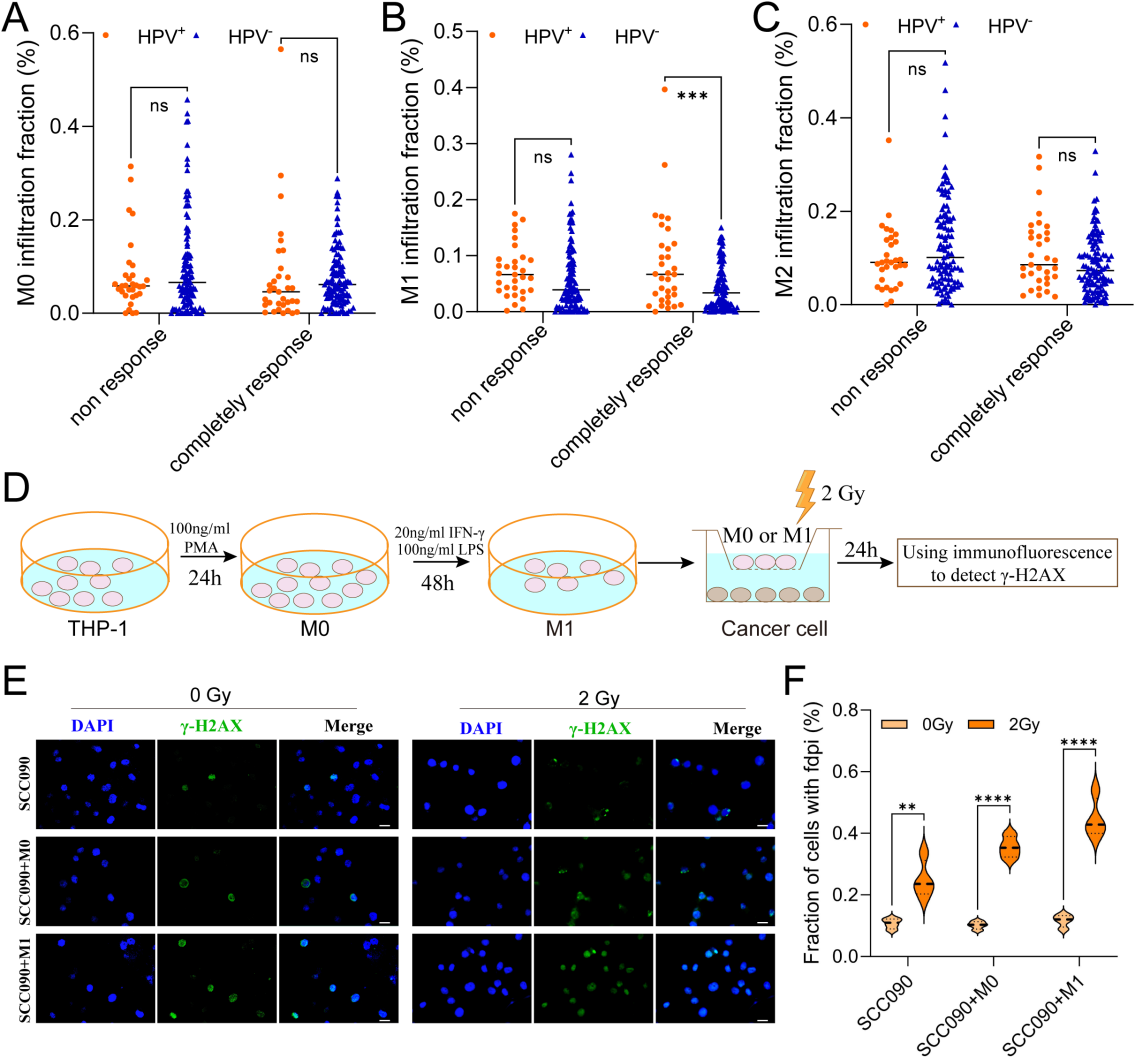
M1 macrophages infiltration promoted the radiosensitivity of HPV^+^ HNSCC. **(A-C)** The relationship between M0, M1 and M2 macrophages infiltration contribute to radiosensitivity of HPV^+^ HNSCC and HPV^-^ HNSCC from TCGA. **(D)** The model showed the steps of functional test. **(E)** Immunofluorescence staining for γ-H2AX foci in different treatment SCC090 cells after 0 Gy or 2 Gy irradiation 24 h (scale bar = 20 μm). **(F)** Quantitation of γ-H2AX foci after irradiation 24 h. ns, not significance. ^**^P < 0.01, ^***^P < 0.001, ^****^P < 0.0001.

This study aims to investigate the influence of TAMs infiltration on the radiosensitivity of HNSCC. Analysis of macrophage infiltration and radiosensitivity data in the TCGA-HNSC cohort showed that, among patients with a complete response, HPV^+^ HNSCC had higher M1 macrophages infiltration compared to HPV^-^ HNSCC (**Figure 2B**). In contrast, no significant differences were observed in the infiltration levels of M0 and M2 macrophages between the two groups (**Figures 2A, C**).

The *in vitro* experimental procedure was illustrated in the schematic diagram (**Figure 2D**). Using co-culture system, SCC090 cells were treated under differently conditions and randomly divided into two groups: one serving as the control group and the other receiving 2 Gy of X-ray irradiation. After 24 h, γ-H2AX levels were measured simultaneously in both groups. The results showed that X-ray irradiation significantly increased γ-H2AX levels compared to the non-irradiated group. Furthermore, this effect was more pronounced in SCC090 cells co-cultured with M1 macrophages, which exhibited even higher γ-H2AX levels (**Figures 2E, F**). This suggested that M1 macrophages were essential for raising the radiosensitivity of HPV^+^ HSNCC.

### MiR-20b of M1 exos elevated the radiosensitivity of HPV^+^ HNSCC

**Figure 3:**
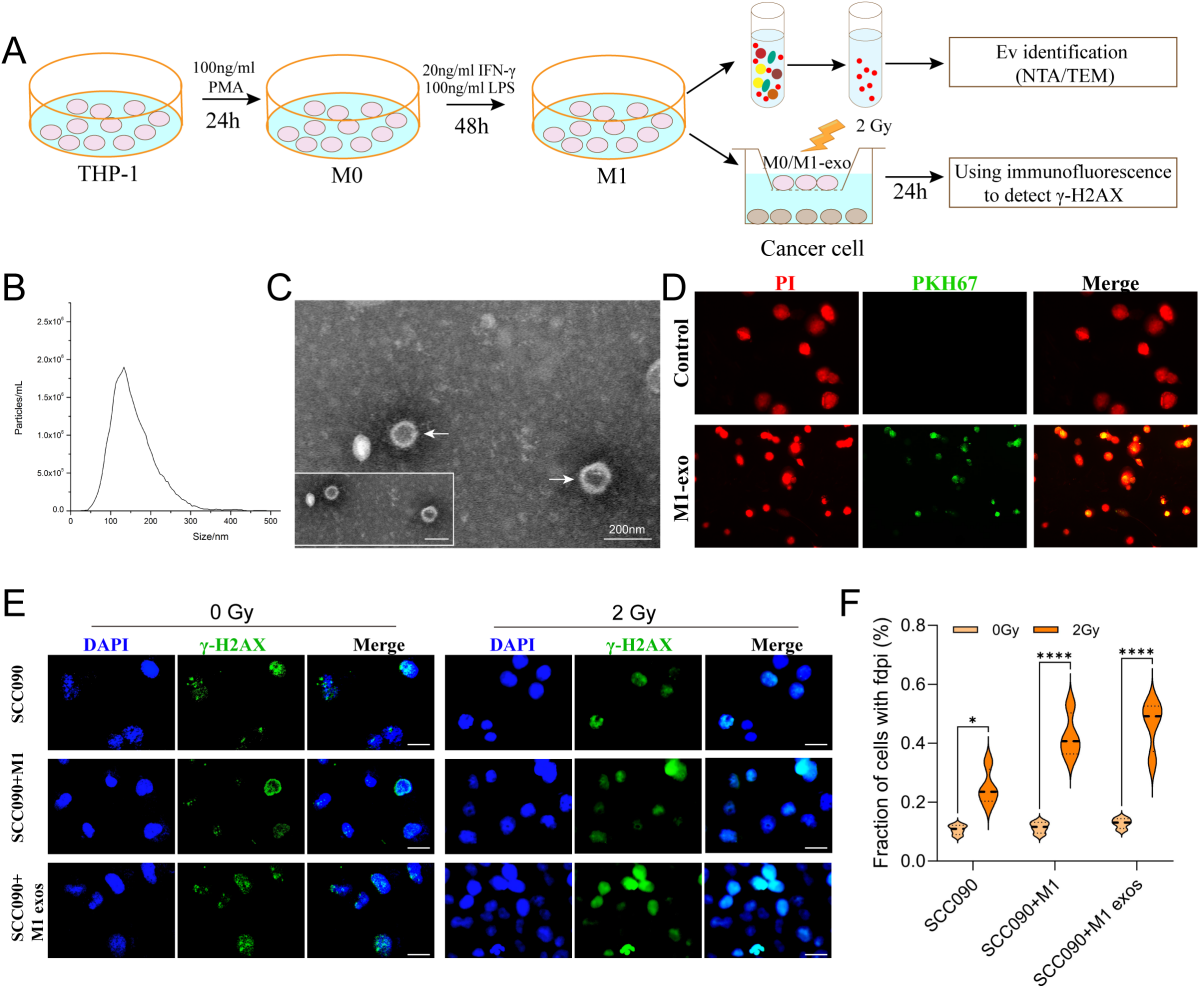
M1 exos promoted radiosensitization of HPV^+^ HNSCC. **(A)** Schematic of M1 exos extraction and M1 macrophages polarization. The detailed description was shown in the Materials and Methods. **(B)** Representative immunofluorescence images of PKH67-labeled M1 exos (green) internalized into SCC090 cells (red). **(C)** TEM image of M1 exos. **(D)** NTA of M1 exos. **(E)** Immunofluorescence staining for γ-H2AX foci in different treatment SCC090 cells after 0 Gy or 2 Gy irradiation 24 h (scale bar = 20 μm). **(F)** Quantitation of γ-H2AX foci 24 h after irradiation. M1 exos, M1 macrophage derived exosome; NTA, nanoparticle tracking analysis; TEM, transmission electron microscopy. ns, not significance; ^*^P < 0.05, ^****^P < 0.0001.

In order to investigate the M1 exos influence the radiosensitivity of SCC090 cells. First, M1 macrophages were induced according to the diagram, and M1 exos were isolated from the cell culture medium using differential ultracentrifugation (**Figure 3A**). NTA analysis revealed high exosome concentrations in the range of 50–150 nm (**Figure 3B**). TEM analysis revealed a characteristic two-disc structure (**Figure 3C**). The expression of CD81 and TSG101 was observed in M1 exos, while calnexin was absent (**Supplementary Figure S2**). This finding corroborated the distinct characteristics of M1 exos. Next, M1 exos were labeled with fluorescent PKH67 and incubated with SCC090 cells, which demonstrated that M1 exos were internalized by SCC090 cells (**Figure 3D**). According to the *in vitro* experimental protocol, SCC090 cells from different treatment groups were randomly divided into control and 2 Gy irradiation groups. The results demonstrated that X-ray irradiation significantly increased γ-H2AX levels in cells, with the most pronounced effect observed in those co-cultured with M1 exos (**Figures 3E, F**).

**Figure 4:**
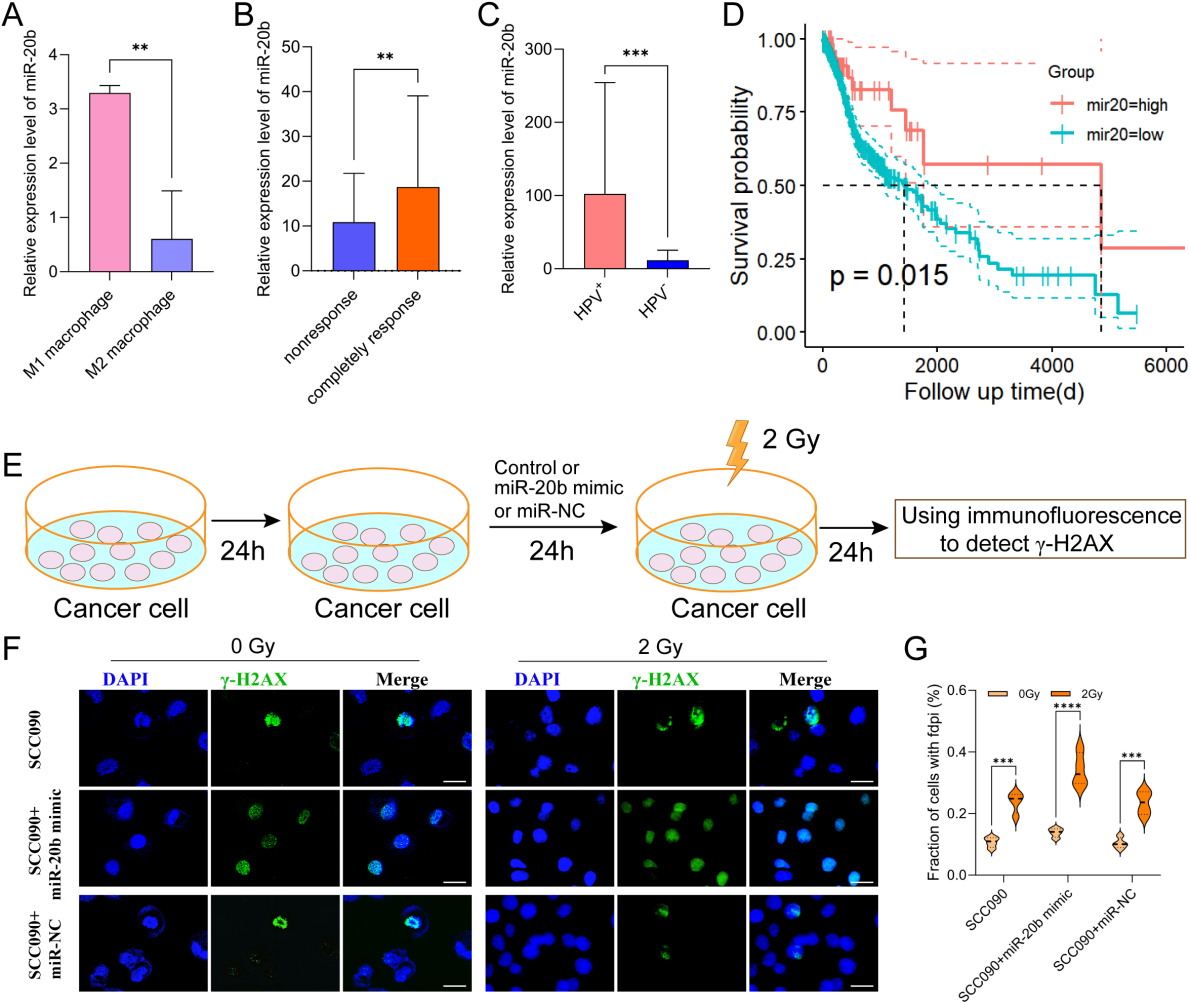
Increased radiosensitivity of HPV^+^ HNSCC by miR-20b. **(A)** The expression of miR-20b in M1 and M2 macrophages by miRNA-seq from GEO. **(B)** MiR-20b expression between complete response group and no response group from TCGA. **(C)** MiR-20b expression between HPV^-^ and HPV^+^ HNSCC from TCGA. **(D)** miR-20b expression and the outcome in HNSCC from TCGA. **(E)** miR-20b mimic transfection flowchart. **(F)** Immunofluorescence staining for γ-H2AX foci in different treatment SCC090 cells after 0 Gy or 2 Gy irradiation 24 h (scale bar = 20 μm). **(G)** Quantitation of γ-H2AX foci after irradiation 24 h. ns, not significant; ^**^P < 0.01, ^***^P < 0.001, ^****^P < 0.0001.

MiR-20b is the miRNA most closely associated with HPV 16 and linked to an improved prognosis in HPV^+^ HSNCC. Therefore, we analyzed the miRNA-seq expression profile data of macrophages from GSE51307 and found that the expression of miR-20b in M1 macrophages was significantly higher than that in M2 macrophages (*p* < 0.01) (**Figure 4A**). Additionally, miRNA data from TCGA indicated that miR-20b was significantly elevated in radiosensitive patients with HNSCC (*p* < 0.01) (**Figure 4B**). Furthermore, compared to HPV^-^ HNSCC, miR-20b levels were significantly higher and positively correlated with a favorable prognosis in HPV^+^ HNSCC compared to HPV^-^ HNSCC (*p* < 0.001) (**Figures 4C, D**).

In order to confirm that M1 exos elevated miR-20b levels in SCC090 cells, we detected the expression level of miR-20b under different co-culture system. The results showed that M1 exos co-cultured with SCC090 significantly increased the level of miR-20b in SCC090 cells compared with SCC090 treated by M0 macrophages or M1 macrophages (**Supplementary Figure S3**). To further investigate the role of miR-20b in radiosensitivity of HPV^+^ HNSCC, the miR-20b mimic was utilized to upregulate the expression of miR-20b in SCC090 cells, as illustrated in the schematic diagram (**Figure 4E**). We successfully upregulated miR-20b expression in SCC090 cells by transfecting with miR-20b mimics (**Supplementary Figure S4**). SCC090 cells to different treatments were randomly assigned to control and 2 Gy X-ray radiation. Results showed that γ-H2AX foci in SCC090 cells treated with miR-20b mimics were significantly increased compared to the control group, and this increase was more pronounced than in the corresponding non-transfected control groups (**Figures 4F, G**). Furthermore, analysis of cell viability 24 hours after irradiation (0 Gy or 2 Gy) demonstrated that transfection with the miR-20b mimic significantly reduced the viability of SCC090 cells following radiotherapy (**Supplementary Figure S5**). We also assessed the effect of miR-20b on various HNSCC cell lines. Using the same transfection approach to overexpress miR-20b in SCC47 and CAL27 cells, we observed that it differentially enhanced their radiosensitivity (**Supplementary Figures S6, S7**). Those results indicated that M1 macrophages played an important role in the sensitization of HPV^+^ HNSCC through exosomal miR-20b.

### CCND1 is a hub gene in the enhancement of radiosensitivity in HPV^+^ HNSCC by miR-20b

**Figure 5:**
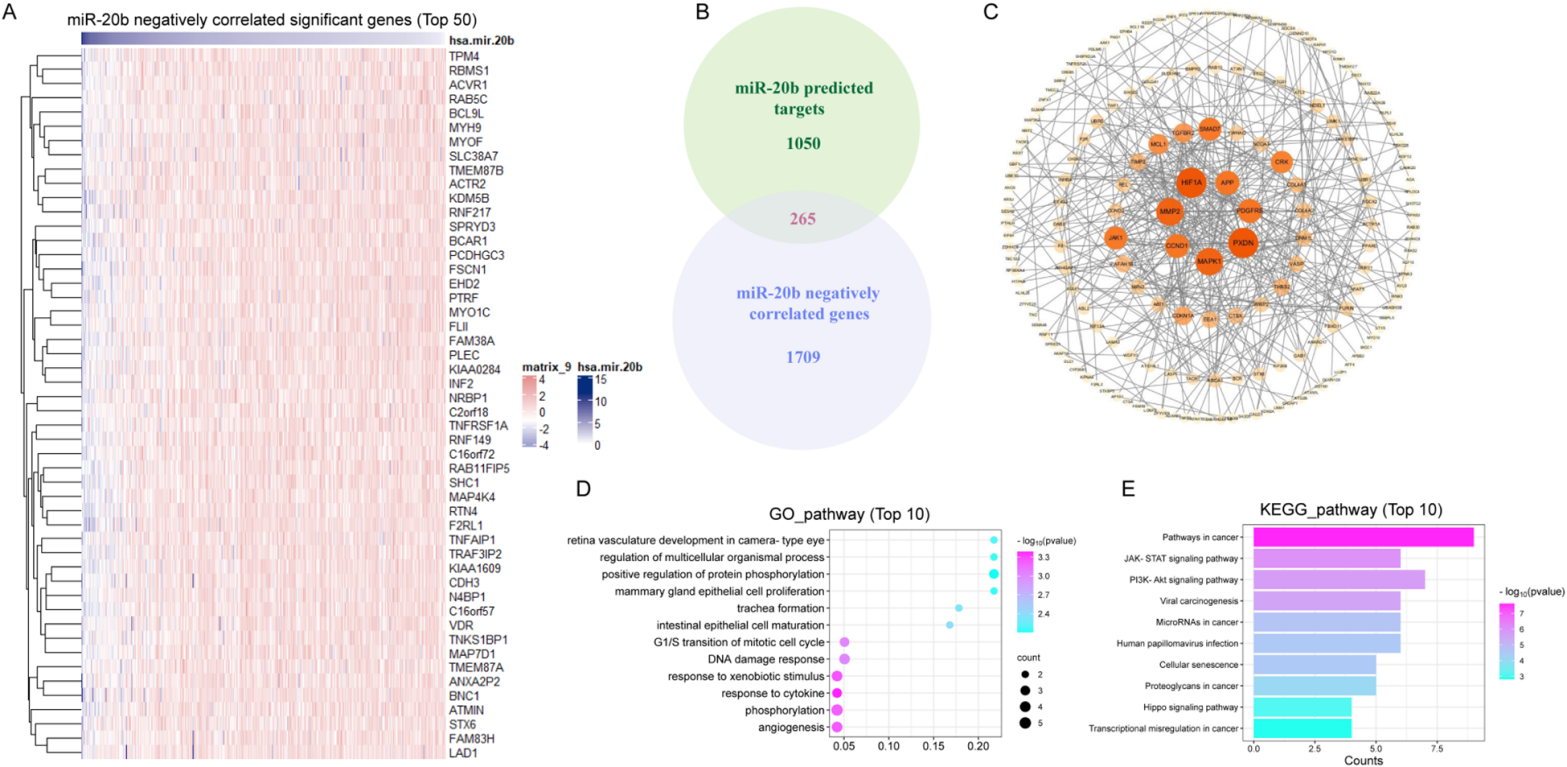
MiR-20b regulates the expression of genes associated with DNA damage repair pathway. **(A)** The genes which negative correlated with miR-20b were analyzed in HNSCC (Top 50); **(B)** The intersection of the genes negatively correlated with miR-20b and the genes predicted to be targeted by miR-20b; **(C)** Cytoscape was used to visualized 265 crossover genes, and the genes with the most crossovers were screened. **(D, E)** The results of mRNA-seq were analyzed by GO enrichment pathway **(D)** and KEGG enrichment pathway **(E)** analyses.

To elucidate the mechanism by which miR-20b enhanced radiosensitivity, we analyzed and identified 1,974 genes that are negatively correlated with miR-20b expression in HNSCC from TCGA. The top 50 genes that exhibit a negative correlation with miR-20b were showed (**Figure 5A**). Target genes of miR-20b were retrieved from the miRDB database, yielding 1,315 genes with a target score > 50. Among the 1,974 genes identified in TCGA, we found 265 overlapping target genes in the miRDB dataset (**Figure 5B**). The 265 genes were visualized using Cytoscape and organized according to their degree scores, revealing seven genes with the highest levels of interaction (**Figure 5C**). To clarify the function of miR-20b target genes, a functional enrichment analysis was conducted on 265 genes. The results of the GO enrichment analysis indicated that miR-20b was significantly associated with cytokine response, DNA damage repair response, and the G1/S transition of the mitotic cell cycle (**Figure 5D**). Additionally, the KEGG enrichment analysis revealed that the target genes were enriched in cancer pathways, as well as the JAK-STAT and PI3K-AKT signaling pathways (**Figure 5E**). These findings suggest that miR-20b target genes are linked to tumorigenesis, cell proliferation, and DNA damage response pathways. Notably, MAPK1, PDGFRB, CCND1, MMP2, HIF1A, and PXDN are all implicated in DNA damage repair and cell cycle-related pathways.

**Figure 6:**
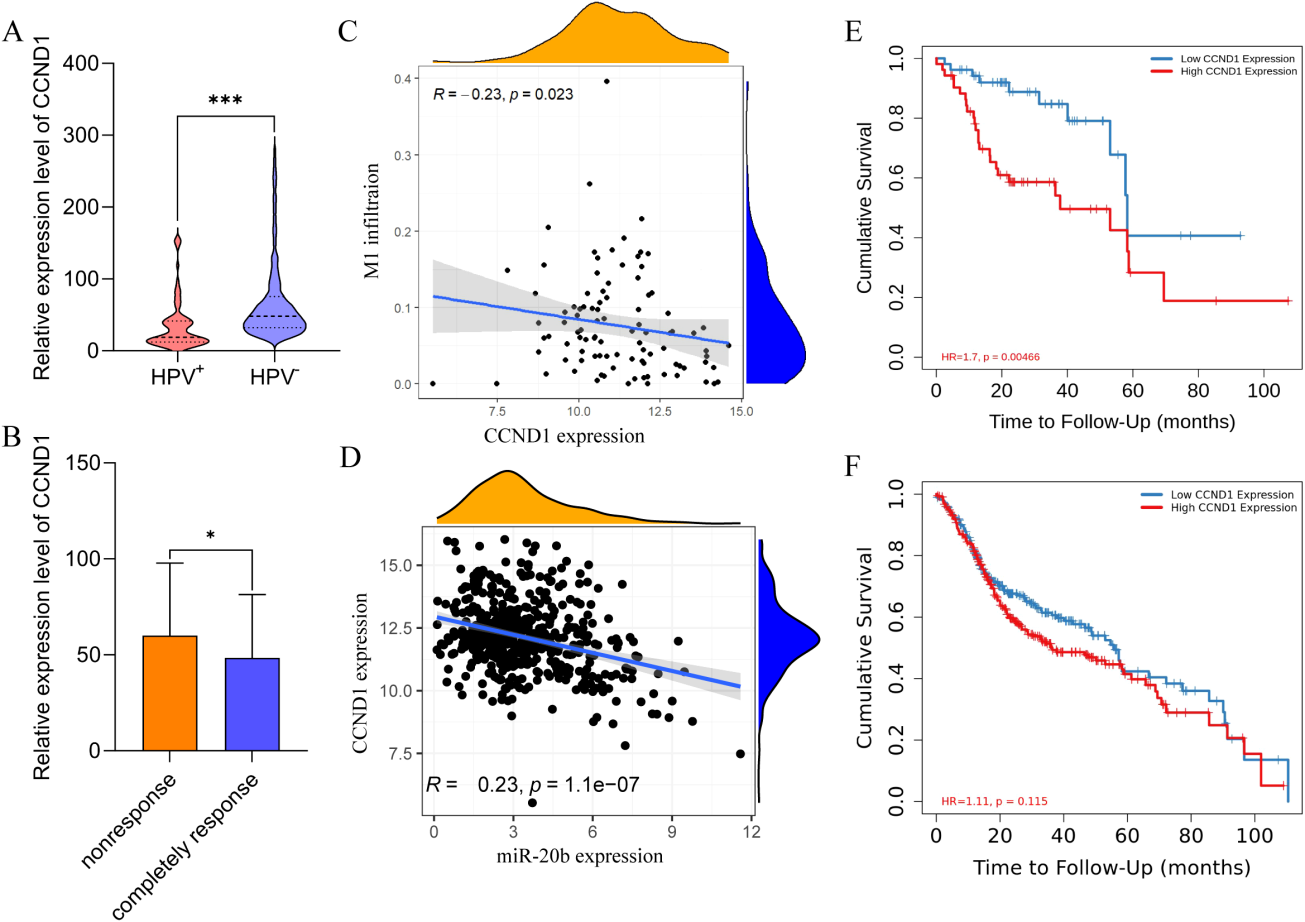
CCND1 is a hub gene for promoting the radiosensitivity of HPV^+^ HNSCC. **(A)** Expression of CCND1 in HPV^+^ HNSCC and HPV^-^ HNSCC from TCGA. **(B)** The expression of CCND1 in radiotherapy-sensitive HNSCC. **(C)** Correlation analysis of CCND1 expression and M1 macrophages infiltration in HPV^+^ HNSCC. **(D)** Correlation analysis of CCND1 and miR-20b expression. **(E, F)** HPV^+^ HNSCC **(E)** and HNSCC **(F)** survival respectively relative to the expression of CCND1. CCND1, cyclin D1. ^*^P < 0.05, ^***^P < 0.001.

To identify the primary target genes of miR-20b that influence the DNA damage repair pathway and cell cycle transition, we analyzed the expression levels of these genes across different HPV statuses and radiotherapy sensitivities. Using Pearson correlation analysis, we assessed the relationship between the target genes, M1 macrophage infiltration, and the expression levels of miR-20b. The results indicated that the expression of CCND1, PDGFRB, HIF1A, PXDN and MMP2 was significantly reduced in the HPV^+^ HNSCC (**Figure 6A**, **Supplementary Figures S8B-E**), and the expression of MAPK1 was no difference in HPV^+^ HNSCC (**Supplementary Figure S8A**). In completely response of radiotherapy cohorts, the expression of CCND1, MAPK1, PDGFRB and PXDN was decreased (**Figure 6B**, **Supplementary Figures S9A, B, D**), and the expression of HIF1A and MMP2 was no effect on radiotherapy sensitivity (**Supplementary Figures S9C, E**). In order to investigate the influence of target genes on M1 macrophage infiltration, correlation analysis revealed that only CCND1 exhibited a negative correlation with M1 macrophage infiltration in HPV^+^ HNSCC (**Figure 6C**). However, other target genes did not show a significant association with M1 macrophage infiltration in either HPV^+^ HNSCC (**Supplementary Figure S10**). Using the same method, a correlation analysis of target genes and miR-20b expression was conducted. The results indicated that all target genes were negatively correlated with miR-20b, consistent with the original findings regarding these target genes (**Figure 6D**, **Supplementary Figure S11**). To further elucidate the relationship between target genes and the outcome of HPV^+^ HNSCC, an analysis using Timer 2.0 revealed that only low expression levels of CCND1 and PXDN were associated with a favorable prognosis for HPV^+^ HNSCC (**Figure 6E**, **Supplementary Figure S12D**). In contrast, the expression levels of the other target genes did not show a significant correlation with prognosis (**Supplementary Figures S12A-C, E**). However, regardless of HPV status, the expression level of CCND1 did not influence the prognosis of HNSCC (**Figure 6F**). In order to further confirm that CCND1 is a key target gene of miR-20b to promote radiotherapy sensitivity, we compared the expression level of CCND1 in SCC090 cells of different treatment groups after radiotherapy, and the results confirmed that the CCND1 level was significantly decreased after M1 macrophages and miR-20b mimic treatment (**Supplementary Figure S13**). Therefore, based on above results, we reasonably speculate that miR-20b primarily alters DNA damage repair and cell cycle-related pathways through the targeted regulation of CCND1 expression.

## Discussion

**Figure 7:**
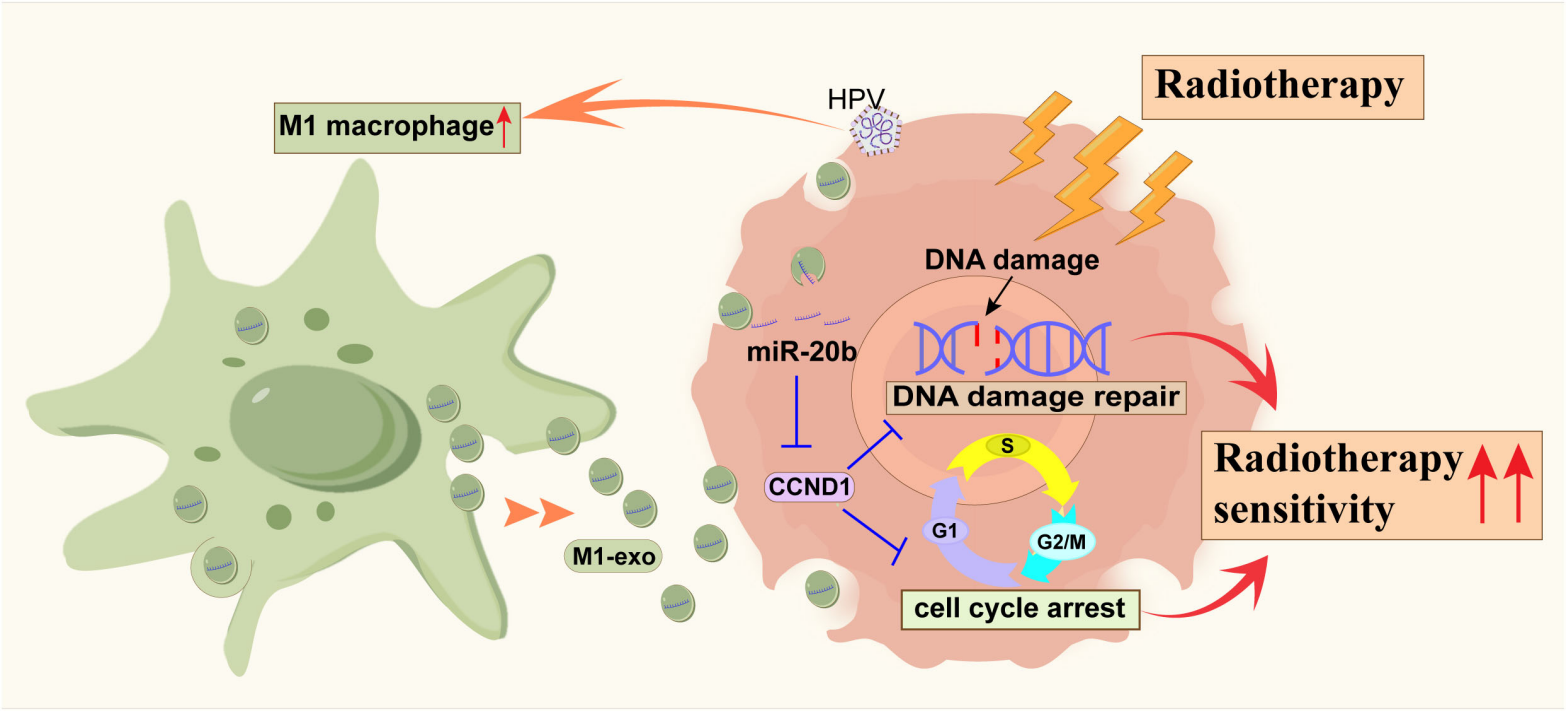
The schematic diagram of miR-20b promoting the radiosensitization of HPV^+^ HNSCC by inhibiting the expression of CCND1.

Our study demonstrates that M1 macrophages enhance radiosensitivity in HPV-positive HNSCC by delivering miR-20b-enriched exosomes to tumor cells. Within these cells, miR-20b potentiates the radiation response by suppressing the DNA damage repair pathway (**Figure 7**). HPV^+^ HNSCC exhibits a better prognosis and greater radiosensitivity compared to HPV^-^ HNSCC (25). Successful cancer therapy is contingent upon the tumor microenvironment (TME) of head and neck squamous cell carcinoma (HNSCC), which is highly diverse and complex (5). As the primary modality for tumor treatment, the efficacy of radiotherapy is influenced by DNA damage repair mechanisms and cell cycle distribution (23, 26). Our findings further support the established model in which HPV enhances radiosensitivity through its encoded oncogenes, which disrupt DNA damage repair pathway, collectively rendering tumor cells more vulnerable to radiation (27, 28).

As the primary immune cells in the tumor microenvironment, macrophages can be polarized into M1 and M2 macrophages following stimulation, which play a “good” or “bad” role in tumor progression (29). With the advancement of nanotechnology, the exchange of information between tumor cells and surrounding cells has been identified through the exosome pathway, including the regulation of macrophage polarization. In laryngeal squamous cell carcinoma, tumor cells induce macrophages to polarize into M2 macrophages via long non-coding RNA HOX transcript antisense RNA contained in exosomes, which diminishes the anti-tumor effect (30). Additionally, our previous results demonstrated that exosomes released by HPV^+^ HNSCC promoted the polarization of mononuclear macrophages to M1 macrophages, improving tumor prognosis (11). In this study, we found that M1 exos attenuated the DNA damage repair pathway in tumor cells, thereby enhancing their sensitivity to radiotherapy.

Exosomes serve as important mediators of intercellular communication and play a role in anti-tumor activity through the delivery of miRNAs (31). Dysregulation of miRNA expression is closely associated with cancer and can either promote or inhibit tumor progression. Studies have demonstrated that miRNAs are transferred to target cells via exosomes, thereby regulating the functions of those target cells (32, 33). Research indicates that miR-20b, a well-established cancer biomarker, plays a crucial role in regulating the cell cycle, proliferation, and apoptosis. It exhibits both anti-tumor and pro-tumor effects in various tumors (34). In breast and prostate cancers, elevated miR-20b promotes tumor proliferation by suppressing phosphatase and tensin homolog (35, 36). Conversely, in bladder cancer, miR-20b suppresses proliferation by inhibiting cyclin-dependent kinases to induce cell cycle arrest (37, 38). This functional duality highlights the context-dependent nature of miR-20b. Although we did not investigate its role in proliferation, our study uncovers a separate mechanism in HNSCC, demonstrating that miR-20b enhances radiosensitivity by disrupting the DNA damage repair pathway.

Through the analysis of target genes and the functional enrichment of miR-20b, as well as the correlation of target genes with M1 macrophage infiltration, radiosensitivity, and HPV status, CCND1 emerged as a prominent gene, which had been identified as the key gene through which miR-20b regulates radiosensitivity in HPV^+^ HNSCC. CCND1, a member of the cyclin family, plays an important role in regulating cell cycle progression and transcription. It is involved in the transition of cells from the G1 phase to the S phase by forming complexes with cell cycle-related kinases, thereby promoting cell proliferation (39, 40). Research has demonstrated that CCND1 serves as a biomarker for tumor phenotype and progression (9). In our study, upregulation of miR-20b enhanced γ-H2AX levels and reduced CCND1 expression following radiotherapy, ultimately suppressing tumor growth. These findings align with the established proliferative role of CCND1 and further support our conclusion.

However, our study has several limitations. Firstly, the limited clinical sample size necessitates future investigation with larger, multi-center cohorts to strengthen the reliability and generalizability of the findings. Secondly, the identification of CCND1 as a target of miR-20b, which was based on bioinformatics analysis, remains unverified. Direct experimental evidence, including genetic gain-/loss-of-function studies and *in vivo* validation, is needed to confirm the regulatory relationship and its functional consequences in the pathway. Future work will be directed toward verifying this axis in a physiological setting and delineating the molecular mechanism by which the miR-20b/CCND1 pathway regulates DNA damage repair.

In summary, based on the observation that increased infiltration of M1 macrophages improves the prognosis of HPV^+^ HNSCC, we demonstrated that high expression levels of exosomal miR-20b enhanced the radiotherapy sensitivity of HPV^+^ HNSCC. M1 macrophages derived exosomal miR-20b by downregulating the expression of CCND1, which inhibits the activation of the DNA damage repair pathway and arrest the cell cycle, thereby increasing the radiotherapy sensitivity of HNSCC.

## Data Availability

The original contributions presented in the study are included in the article/**Supplementary Material**. Further inquiries can be directed to the corresponding authors.
