# Supplementary material for: M1 macrophage-derived exosomal miR-20b promotes radiosensitization via CCND1 in HPV+ HNSCC
Source: Front Oncol. 2025 Nov 27;15:1693487. doi: 10.3389/fonc.2025.1693487 (PMC12695572; doi:10.3389/fonc.2025.1693487)
Supplement: Supplementary file 1 [file DataSheet1.docx]

# Supplementary data

#
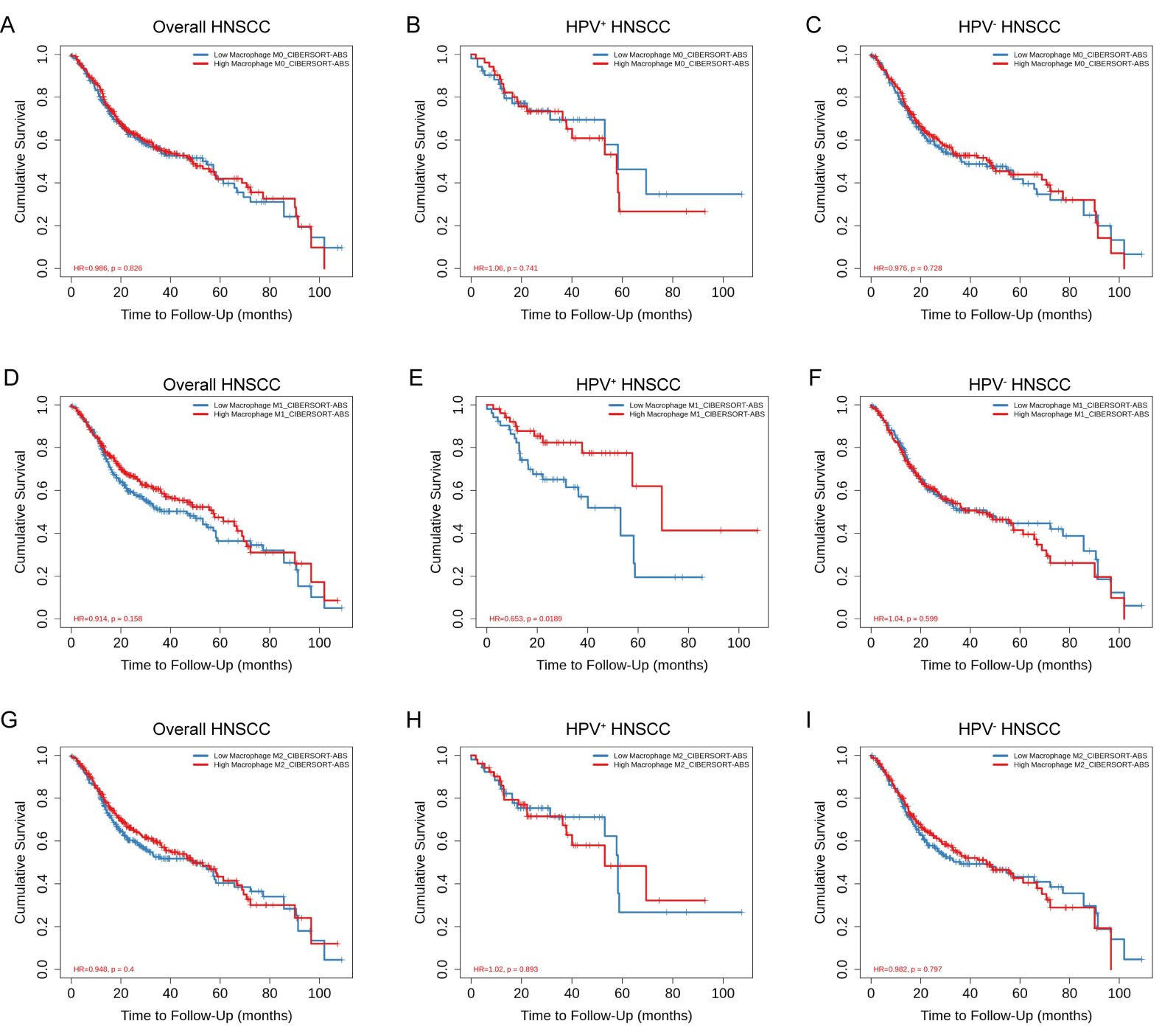
Fig. S1 Prognostic significance of macrophages assessed in HPV^+^ and HPV^-^ HNSCC.

(A-C) Overall, HPV^+^ and HPV^-^ HNSCC survival relative to M0 macrophages. (D-F) Overall, HPV^+^ and HPV^-^ HNSCC survival relative to M1 macrophages. (G-I) Overall, HPV^+^ and HPV^-^ HNSCC survival relative to M2 macrophages. HPV^+^ HNSCC (n = 97), HPV^-^ HNSCC (n = 421).


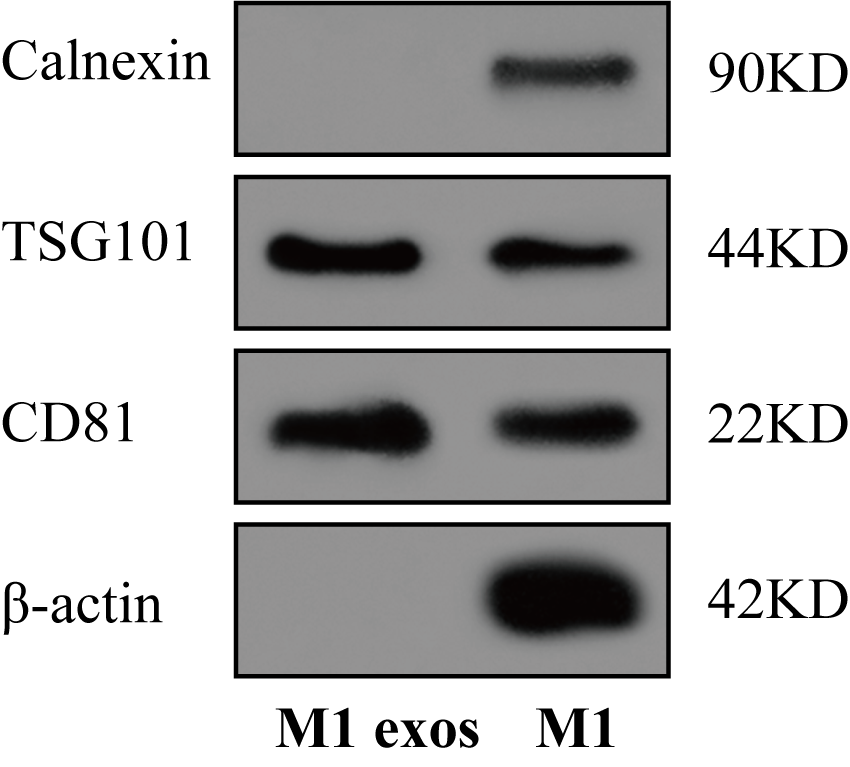


# Fig. S2 Analysis of exosomal proteins using Western blot.

The proteins of CD81, TSG101, Calnexin and β-actin in M1 exosomes and M1 macrophages by Western blot.


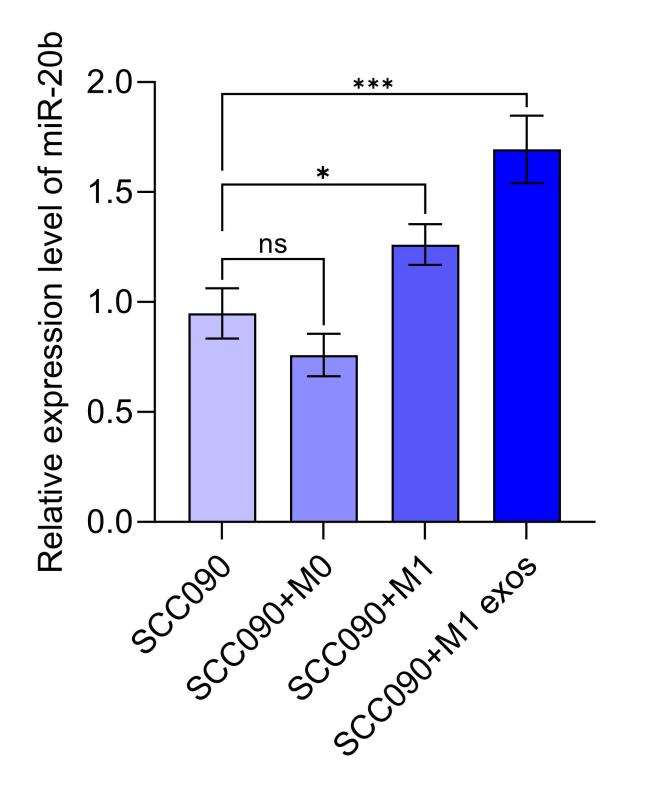


# Fig. S3 M1 exos increased the level of miR-20b in SCC090 cells as detected by qRT-PCR.

ns, not significance; ^*^*P* < 0.05, ^**^*P* < 0.01, ^***^*P* < 0.001, ^****^*P* < 0.0001.


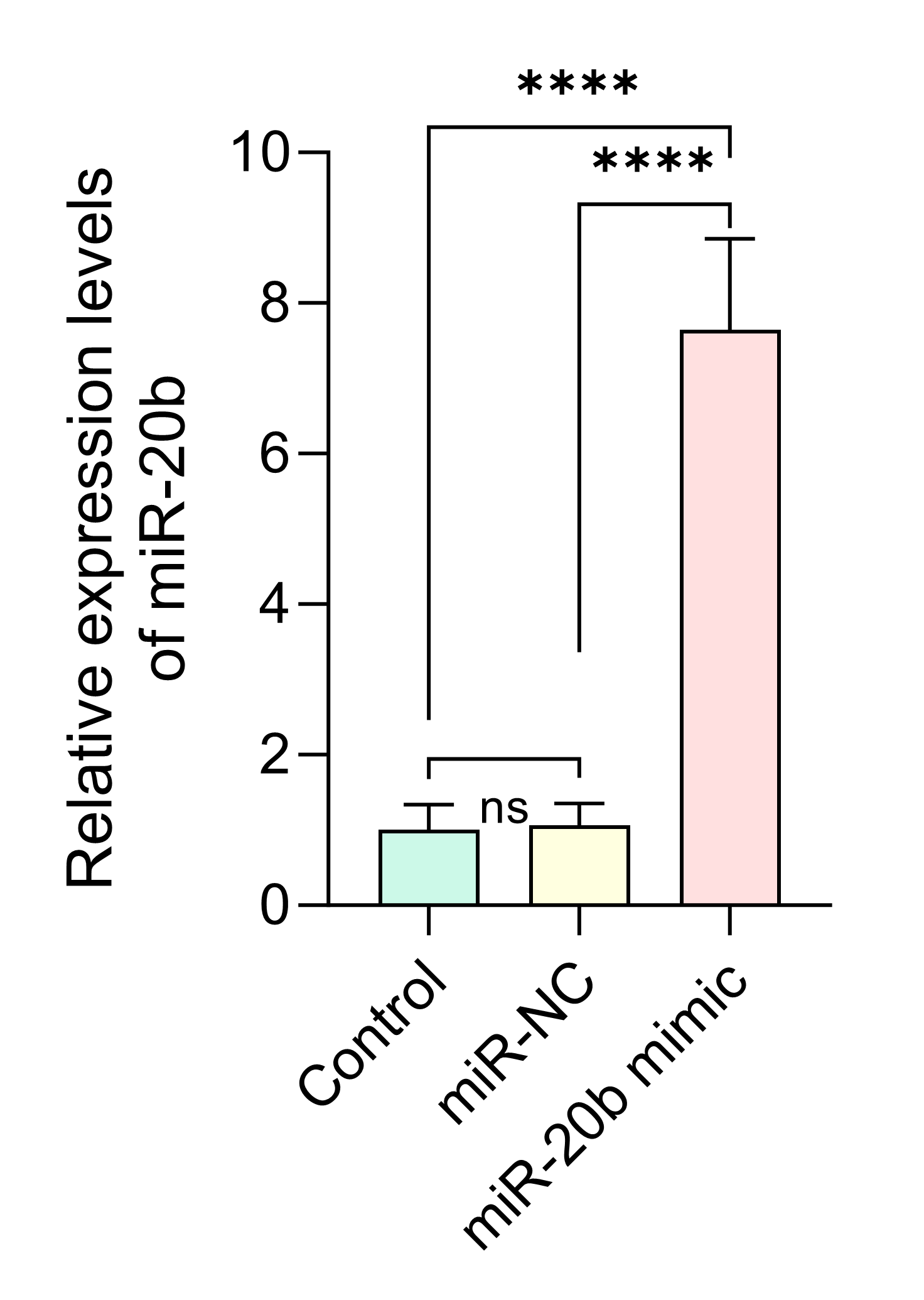


# Fig. S4 The expression level of miR-20b was detected after transfection by qRT-PCR.

ns, not significance; ^*^*P* < 0.05, ^**^*P* < 0.01, ^***^*P* < 0.001, ^****^*P* < 0.0001.


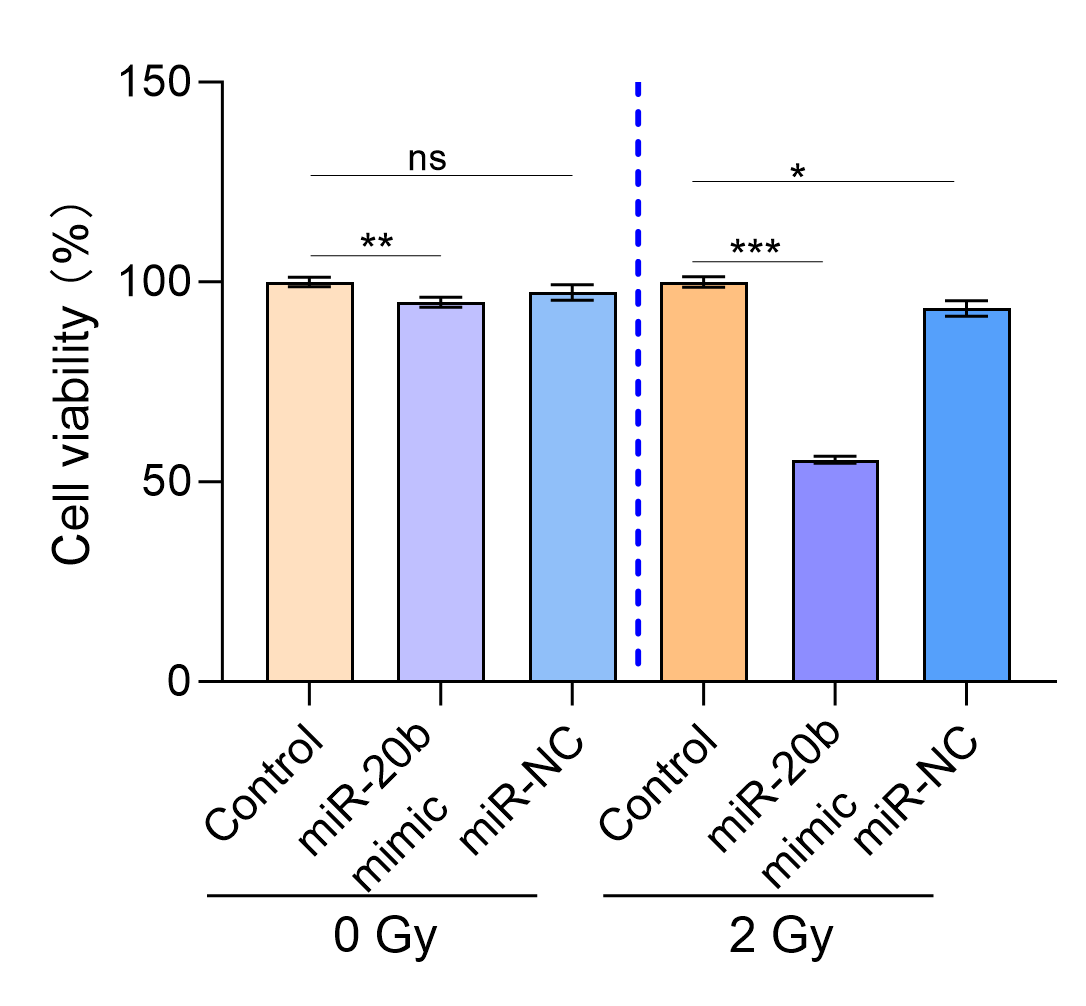


# Fig. S5 The viability of SCC090 cells in various treatment groups was assessed 24 hours after radiotherapy using the CCK-8 assay.

ns, not significance; ^*^*P* < 0.05, ^**^*P* < 0.01, ^***^*P* < 0.001, ^****^*P* < 0.0001.


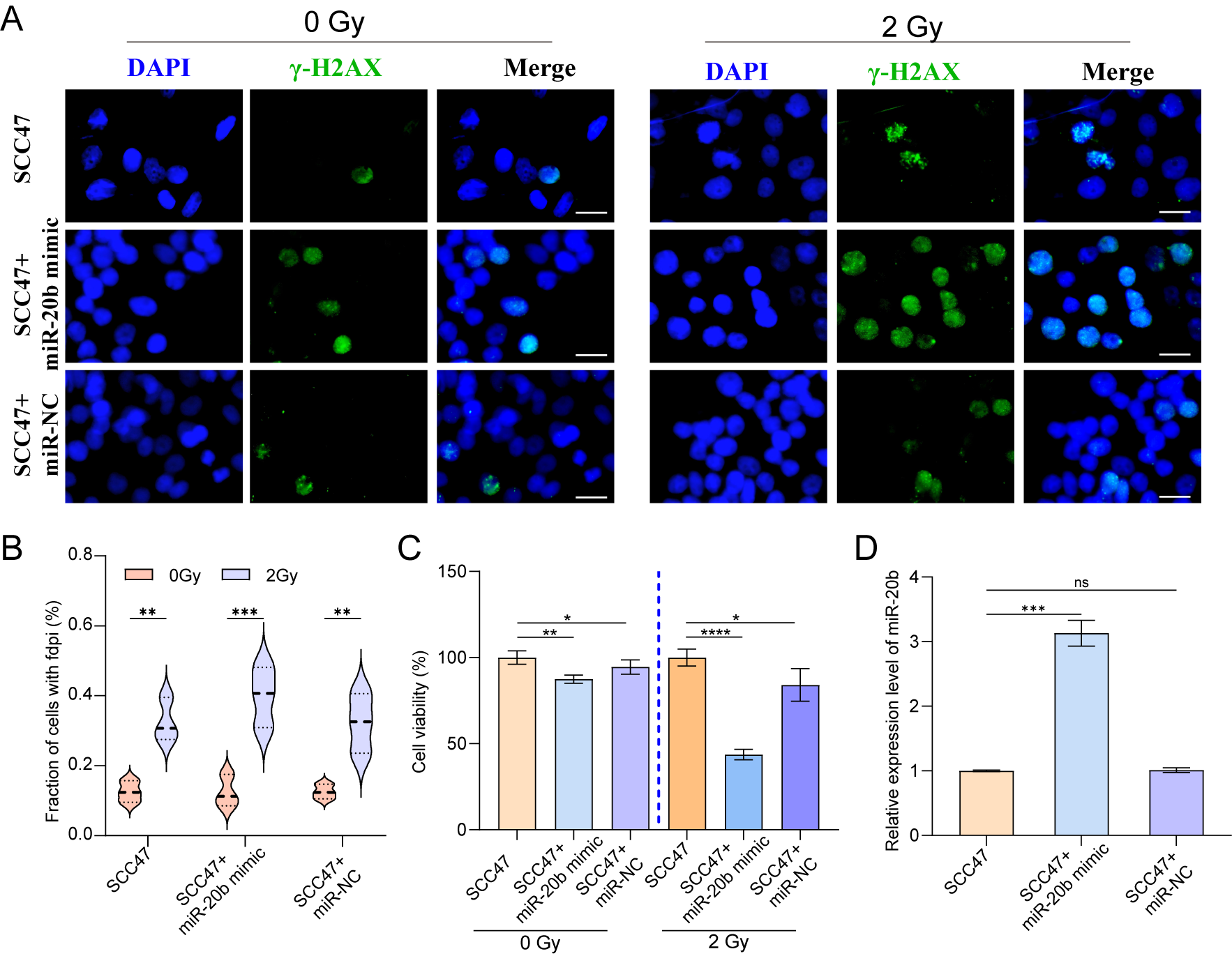


# Fig. S6 MiR-20b enhanced the radiosensitivity of SCC47 cells.

1. Immunofluorescence staining for γ-H2AX foci in different treatment SCC47 cells after 0 Gy or 2 Gy irradiation 24 h (scale bar = 20 μm). (B) Quantitation of γ-H2AX foci after irradiation 24 h. (C) The viability of SCC47 cells in various treatment groups was determined using the CCK-8 assay. (D) The expression level of miR-20b was detected after transfection by qRT-PCR. ns, not significance; ^*^*P* < 0.05, ^**^*P* < 0.01, ^***^*P* < 0.001, ^****^*P* < 0.0001.


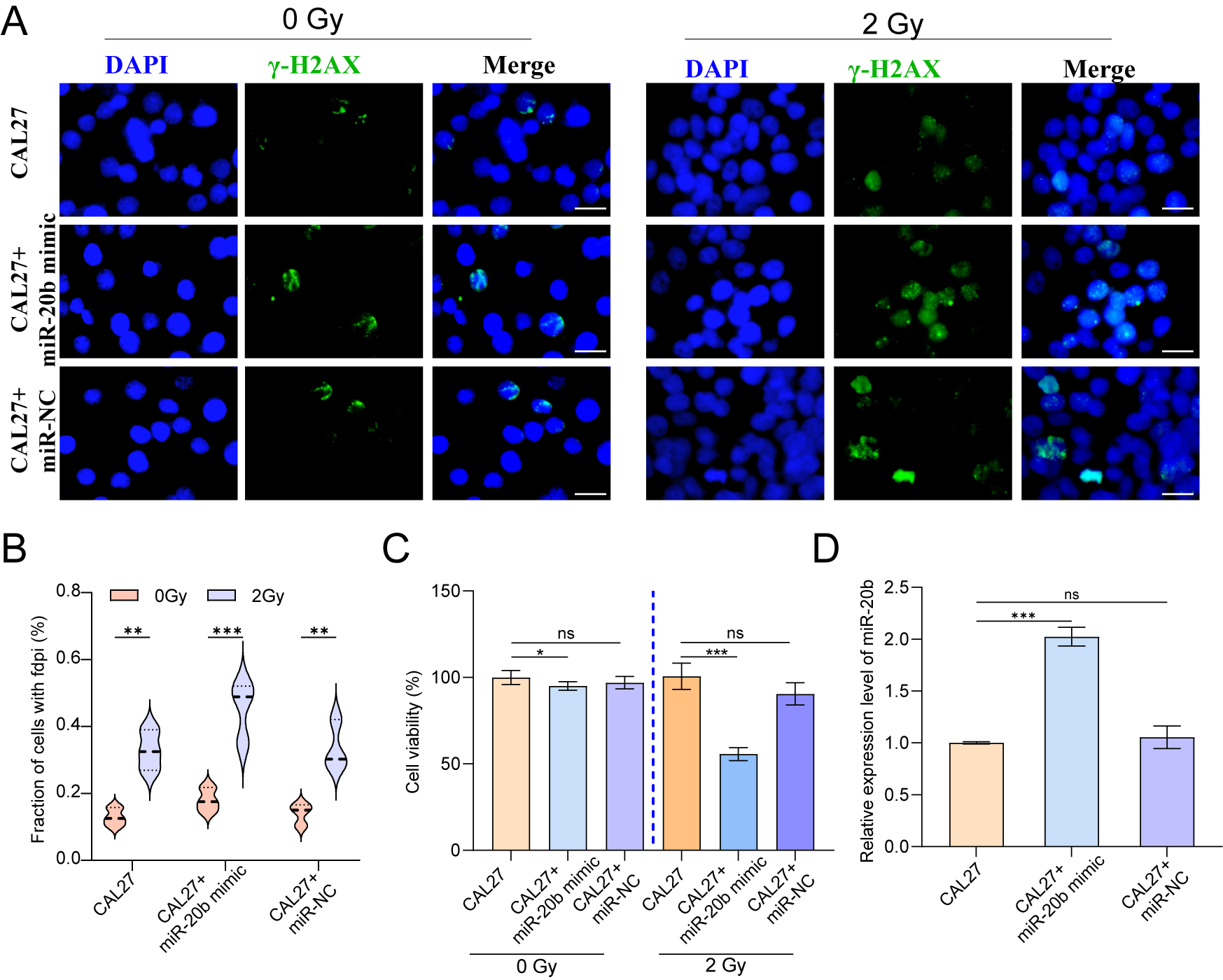


# Fig. S7 MiR-20b enhanced the radiosensitivity of CAL27 cells.

1. Immunofluorescence staining for γ-H2AX foci in different treatment CAL27 cells after 0 Gy or 2 Gy irradiation 24 h (scale bar = 20 μm). (B) Quantitation of γ-H2AX foci after irradiation 24 h. (C) The viability of CAL27 cells in various treatment groups was determined using the CCK-8 assay. (D) The expression level of miR-20b was detected after transfection by qRT-PCR. ns, not significance; ^*^*P* < 0.05, ^**^*P* < 0.01, ^***^*P* < 0.001, ^****^*P* < 0.0001.


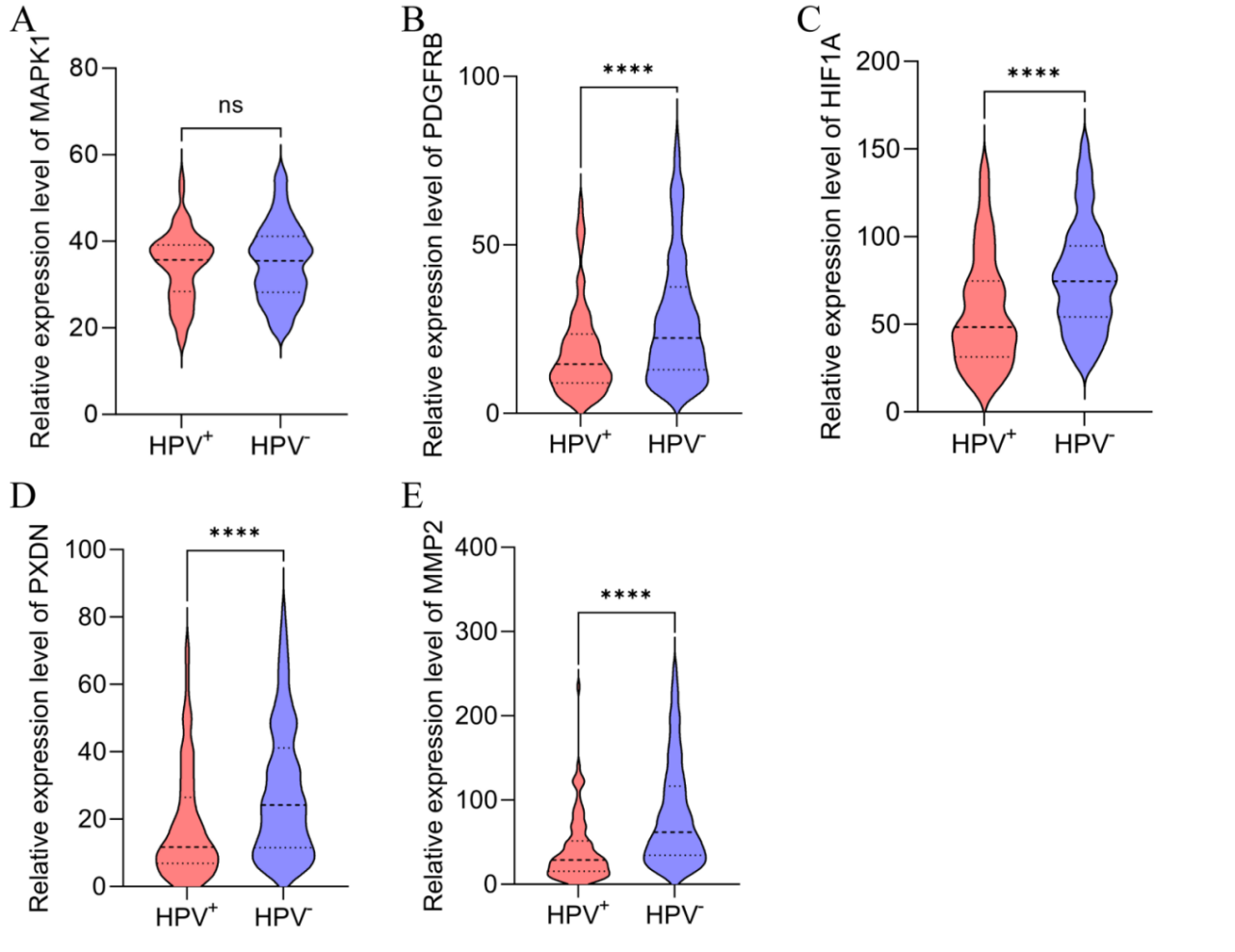


# Fig. S8 The expression level of target genes in HPV^+^ and HPV^-^ HNSCC.

(A-E) The expression of MAPK1 (A), PDGFRB (B), HIF1A (C), PXDN (D) and MMP2 (E) in HPV^+^ HNSCC and HPV^-^ HNSCC. ns, not significance; ^*^*P* < 0.05, ^**^*P* < 0.01, ^***^*P* < 0.001, ^****^*P* < 0.0001.


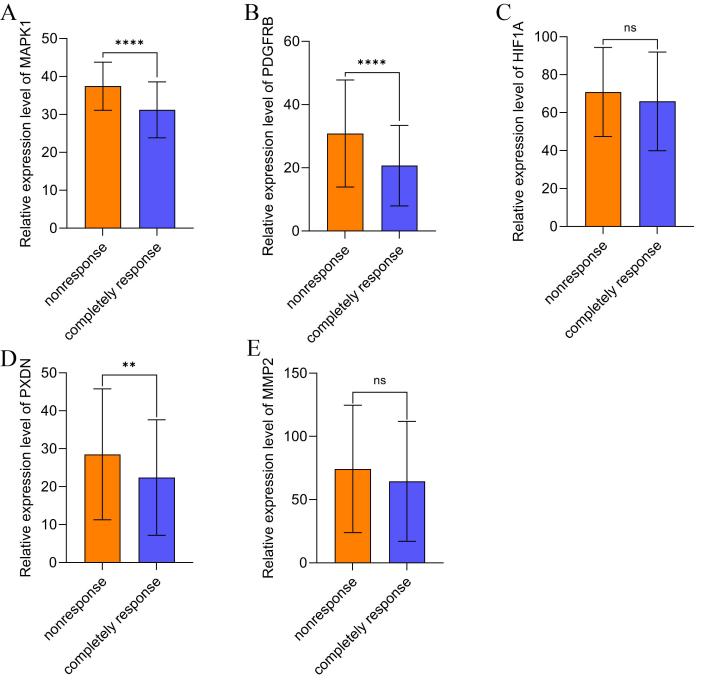


# Fig. S9 The expression of target genes in HNSCC.

(A-E) The expression of MAPK1 (A), PDGFRB (B), HIF1A (C), PXDN (D) and MMP2 (E) in HNSCC with varying sensitivities to radiotherapy. ns, not significance; ^*^*P* < 0.05, ^**^*P* < 0.01, ^***^*P* < 0.001, ^****^*P* < 0.0001.


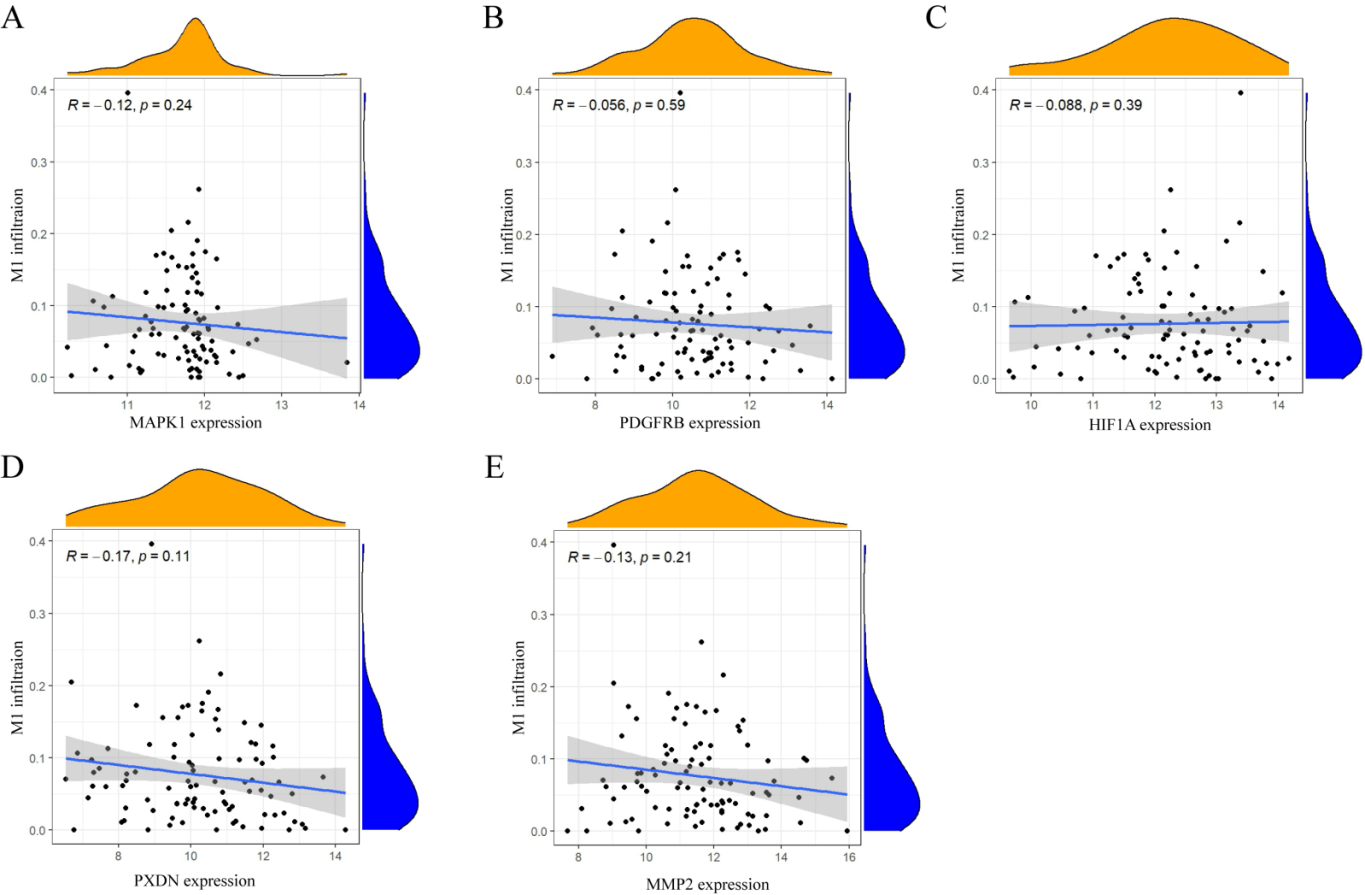


# Fig. S10 Correlation analysis of the expression of target genes and M1 macrophages.

(A-E) Correlation analysis of the expression levels of MAPK1 (A), PDGFRB (B), HIF1A (C), PXDN (D) and MMP2 (E) with the infilitration of M1 macrophages in HNSCC.


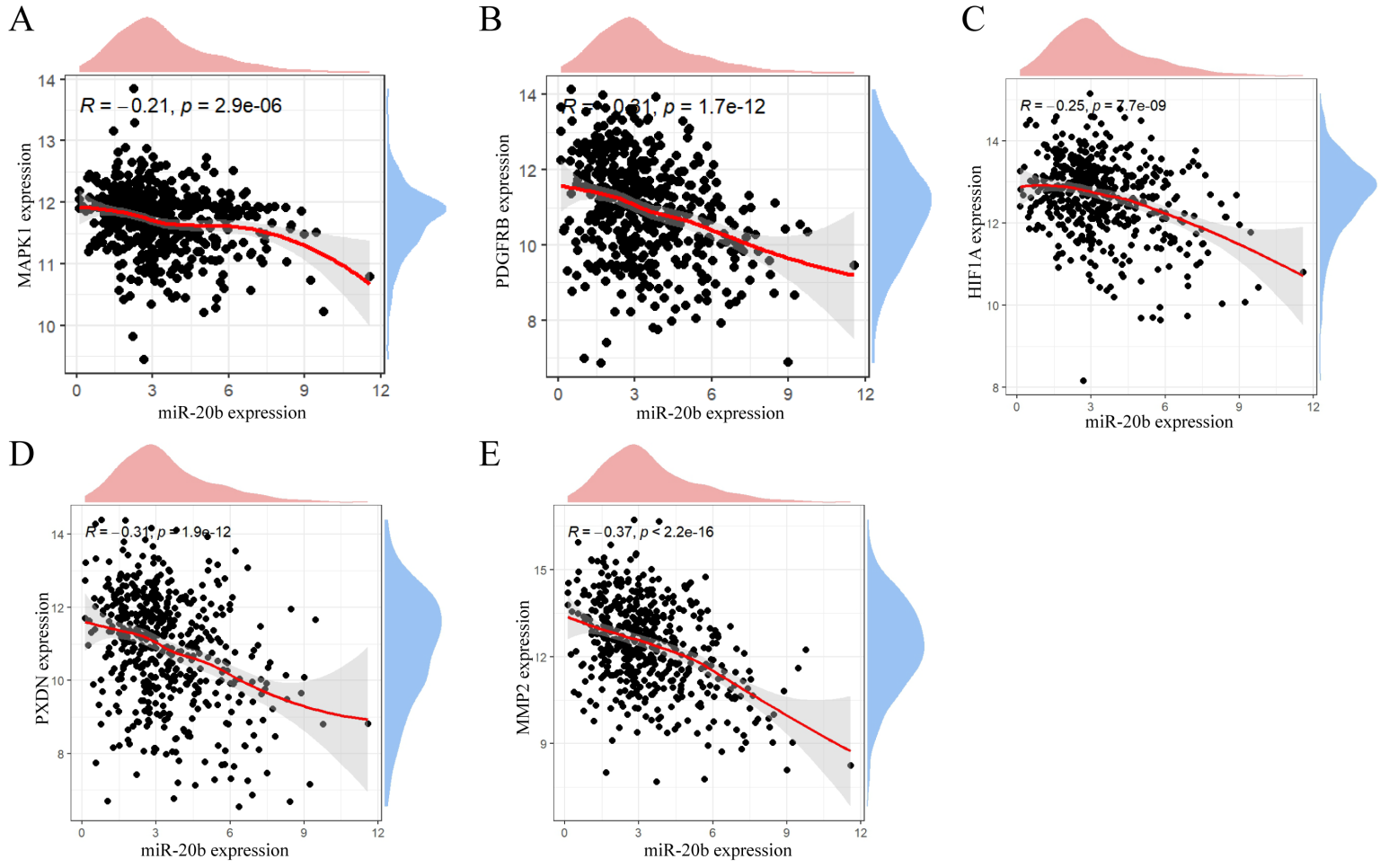


# Fig. S11 Correlation analysis of the expression of target genes and miR-20b.

(A-E) Correlation analysis of the expression levels of MAPK1 (A), PDGFRB (B), HIF1A (C), PXDN (D) and MMP2 (E) with miR-20b in HNSCC.


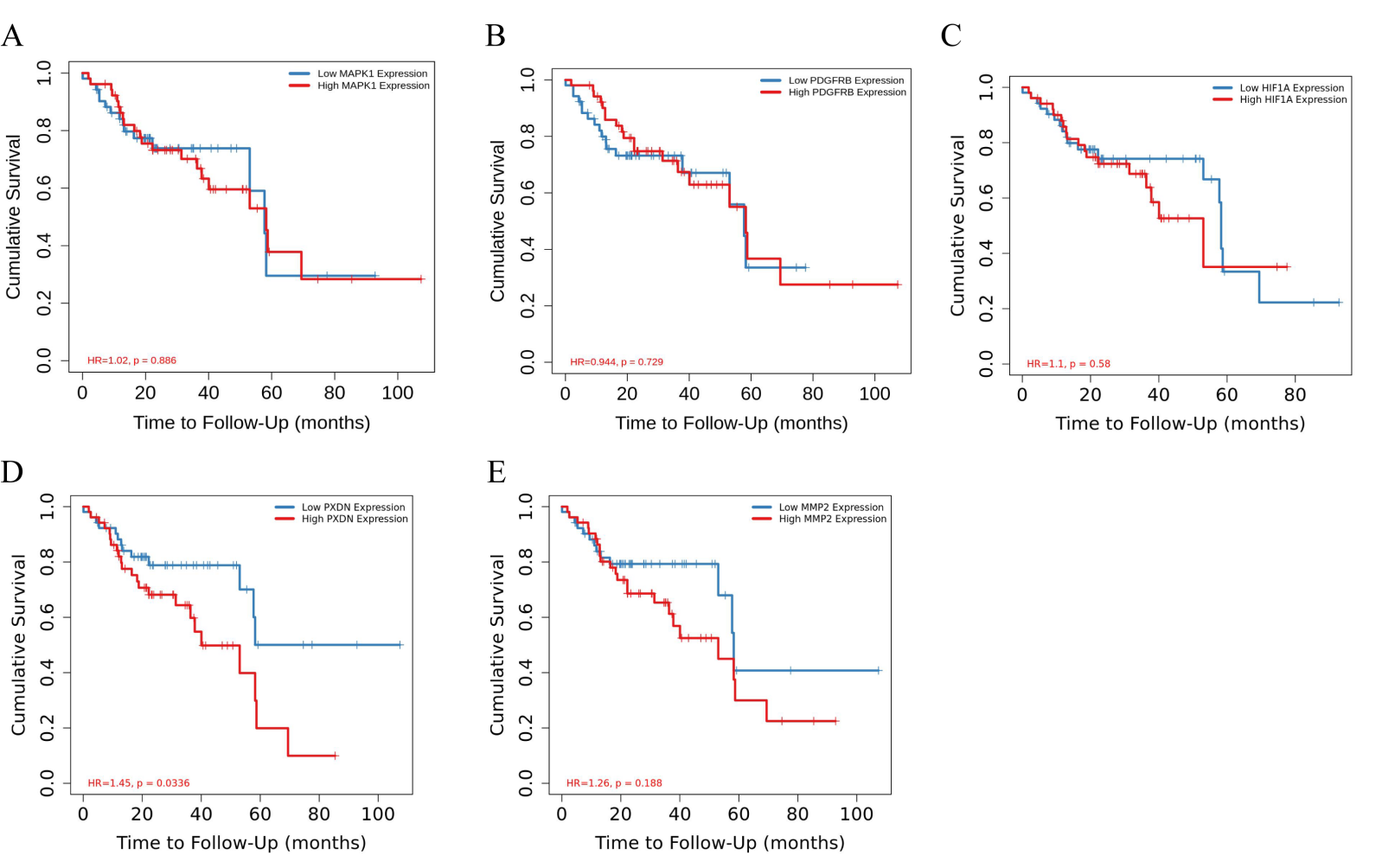


# Fig. S12 The influence of target genes on the outcome of HPV^+^ HNSCC.

(A-E) The effect of the expression levels of MAPK1 (A), PDGFRB (B), HIF1A (C), PXDN (D) and MMP2 (E) on the outcome of HPV^+^ HNSCC.


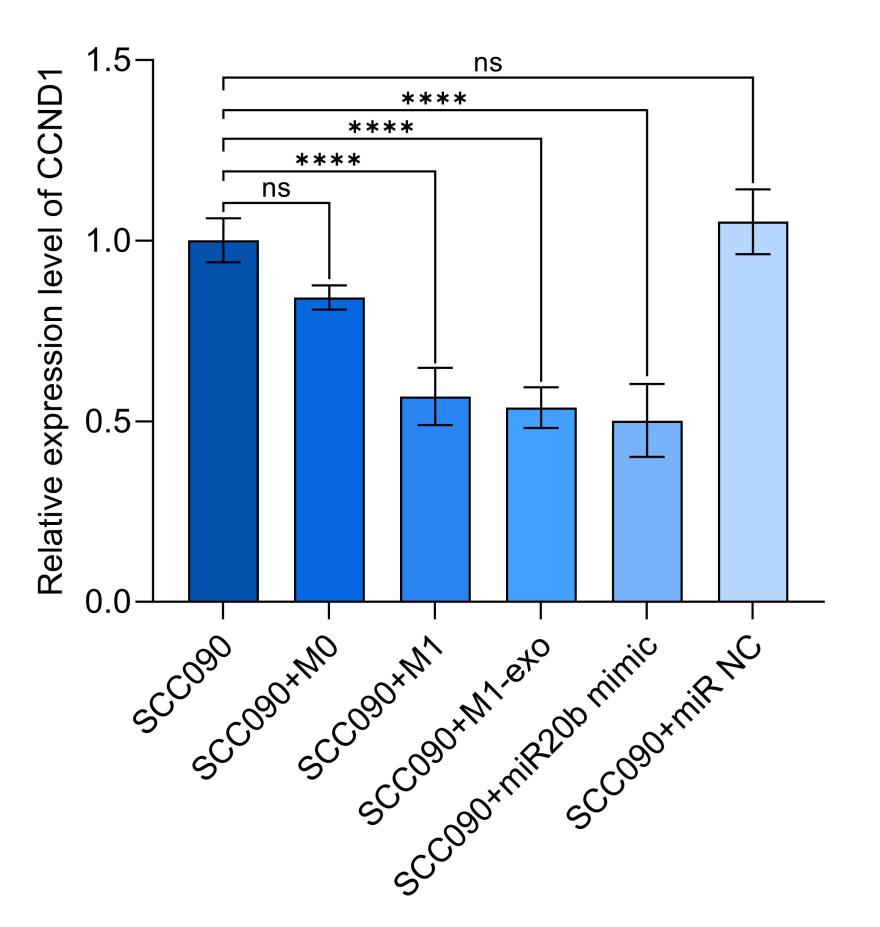


# Fig. S13 The expression level of CCND1 in SCC090 after irradiation was detected by qRT-PCR.

ns, not significance; ^*^*P* < 0.05, ^**^*P* < 0.01, ^***^*P* < 0.001, ^****^*P* < 0.0001.
