# Supplementary material for: M1 macrophage-derived exosomal miR-20b promotes radiosensitization via CCND1 in HPV+ HNSCC
Source: Front Oncol. 2025 Nov 27;15:1693487. doi: 10.3389/fonc.2025.1693487 (PMC12695572; doi:10.3389/fonc.2025.1693487)
Supplement: Supplementary file 2 [file Table1.docx]

| **Table S1. The clinical characteristics.** | | | | |
| --- | --- | --- | --- | --- |
| **Characteristic** | HPV^+^ HNSCC (n = 10) |  | HPV^-^ HNSCC (n = 15) | *P* |
| **Gender** |  |  |  | 0.48 |
| Male | 6 |  | 11 |  |
| Femal | 4 |  | 4 |  |
| **Mean age (years)** | 57.6 |  | 57.7 |  |
| **Smoking** |  |  |  | 0.45 |
| ≧20 | 4 |  | 6 |  |
| <20 | 4 |  | 3 |  |
| never | 2 |  | 6 |  |
| **Alcohol** |  |  |  | 0.43 |
| ≧20 | 3 |  | 2 |  |
| <20 | 1 |  | 4 |  |
| never | 6 |  | 9 |  |
| **Tumor position** |  |  |  | **0.027^*^** |
| Base of tongue | 6 |  | 2 |  |
| Buccal mucosa | 1 |  | 4 |  |
| Floor of mouth | 1 |  | 2 |  |
| Gingiva | 0 |  | 3 |  |
| Palatal | 2 |  | 0 |  |
| Lip region | 0 |  | 3 |  |
| Tongue | 0 |  | 3 |  |
| **TNM stage** |  |  |  | 0.62 |
| I-II | 5 |  | 9 |  |
| III-IV | 5 |  | 6 |  |
| **Differentiztion** |  |  |  | 0.79 |
| Well | 2 |  | 2 |  |
| Moderate | 4 |  | 8 |  |
| Poor | 4 |  | 5 |  |
| *P* value were calculated by Chi-squared test. | | | | |

# **Table S2. Primer sequences for reverse transcription quantitative polymerase chain reaction.**

| Gene | Primer sequence |
| --- | --- |
| miR-20b-5p specific stem-loop | RT5’-GTCGTATCCAGTGCAGGGTCCGAGGTATTCGCACTGGATACGACCTACCT-3’ |
| miR-20b-5p forward | 5’-GCGCAAAGTGCTCATAGTGC-3’ |
| miR-20b-5p reverse | 5’-AGTGCAGGGTCCGAGGTATT-3’ |
| U6 forward | 5’-CTCGCTTCGGCAGCACA-3’ |
| U6 reverse | 5’-AACGCTTCACGAATTTGCGT-3’ |
| CCND1 forward | GAAGGAGACCATCCCCCTGA |
| CCND1 reverse | CAATGAAATCGTGCGGGGTC |
| GADPH forward | GTCTCCTCTGACTTCAACAGCG |
| GADPH reverse | ACCACCCTGTTGCTGTAGCCAA |
